# Erythropoietin Promotes Infection Resolution and Lowers Antibiotic Requirements in *E. coli-* and *S. aureus*-Initiated Infections

**DOI:** 10.3389/fimmu.2021.658715

**Published:** 2021-04-13

**Authors:** Feihong Liang, Huiting Guan, Wenhua Li, Xue Zhang, Tingting Liu, Yu Liu, Jie Mei, Cheng Jiang, Fengxue Zhang, Bangwei Luo, Zhiren Zhang

**Affiliations:** ^1^ Research Center for Integrative Medicine of Guangzhou University of Chinese Medicine, Guangzhou, China; ^2^ Institute of Immunology, Army Medical University, Chongqing, China; ^3^ Department of Respiratory Medicine, The First Affiliated Hospital of Guangzhou University of Chinese Medicine, Guangzhou, China

**Keywords:** erythropoietin, erythropoietin receptor, macrophage, phagocyte, infection

## Abstract

Endogenous mechanisms underlying bacterial infection resolution are essential for the development of novel therapies for the treatment of inflammation caused by infection without unwanted side effects. Herein, we found that erythropoietin (EPO) promoted the resolution and enhanced antibiotic actions in *Escherichia coli* (*E. coli*)- and *Staphylococcus aureus* (*S. aureus*)-initiated infections. Levels of peritoneal EPO and macrophage erythropoietin receptor (EPOR) were elevated in self-limited *E. coli-*initiated peritonitis. Myeloid-specific EPOR-deficient mice exhibited an impaired inflammatory resolution and exogenous EPO enhanced this resolution in self-limited infections. Mechanistically, EPO increased macrophage clearance of bacteria *via* peroxisome proliferator-activated receptor γ (PPARγ)-induced CD36. Moreover, EPO ameliorated inflammation and increased the actions of ciprofloxacin and vancomycin in resolution-delayed *E. coli*- and *S. aureus*-initiated infections. Collectively, macrophage EPO signaling is temporally induced during infections. EPO is anti-phlogistic, increases engulfment, promotes infection resolution, and lowers antibiotic requirements.

## Introduction

Bacterial infections are one of the leading causes of mortality worldwide and have become a pressing public health concern. While the customary approach for treating bacterial infections is administration of antibiotics, the global rise of multidrug-resistant bacteria has attracted new interest in developing novel strategies to treat bacterial infections (1–3).

Host-directed therapies for the treatment of bacterial infections act on the host rather than the pathogen and are intended for use as adjunctive treatments together with conventional antimicrobial drugs, particularly in the face of increasing antibiotic resistance (3, 4). In response to infection, the host mounts inflammatory responses within seconds of pathogen detection to protect the injured host. However, an over-inflammatory cytokine storm response may be responsible for severe immunopathology. Endogenous mechanisms to resolve inflammation are therefore induced to balance antimicrobial activity and tissue damage (5). Recently, the elucidation of the temporal lipid mediator class switching from the generation of proinflammatory mediators to the biosynthesis of lipoxins and specialized proresolving mediators demonstrates that inflammation resolution is an active response (6). Proresolving mediators are produced locally and enhance bacterial killing and clearance along with inhibiting inflammation and promoting tissue regeneration (6–9). Therefore, novel mediators produced in self-resolving infections to promote infection resolution are of great interest and may provide an alternative therapy to antibiotics for the treatment of bacterial infections (10).

Erythropoietin (EPO) is a glycoprotein hormone well-known for its erythropoietic effects and has been widely applied for renal anemia treatment in the clinic. EPO stimulates erythroid progenitor cell proliferation and differentiation in the bone marrow by binding to the EPO receptor (EPOR) (11). However, increasing evidence shows that various non-hematopoietic cells also express EPOR, including macrophages that play an essential role in inflammatory responses (12, 13). We and others have shown that EPO and EPOR are locally upregulated following inflammation/injury and EPO exerts cyto-protective and anti-inflammatory activities (12, 14–19). Moreover, recently we have shown that EPO enhanced macrophage clearance of apoptotic cells can promote inflammation resolution in sterile peritonitis (12, 20). However, the role of EPO in bacterial infection resolution remains to be explored. Herein, we found that EPO promoted infection resolution and lowed the antibiotic requirements of *Escherichia coli* (*E. coli*)- and *Staphylococcus aureus* (*S. aureus*)-initiated infections.

## Methods 

### Bacterial Strains and Growth Conditions

*E. coli* serotype O6: K2: H1 was grown in Luria-Bertani (LB) medium, while *S. aureus* ATCC25923 was grown in brain heart infusion (BHI) broth, both at 37°C with shaking at 220 rpm. Bacteria were washed twice with PBS before inoculation into the mouse peritoneum and enumerated by plating for viable c.f.u. on LB or BHI agar plates.

### Animals

Wild-type C57/BL6 mice were used. Epor^loxp^/^loxp^ mice on a Sv129/C57/BL6 background were backcrossed with C57/BL6 mice for more than ten generations. PPARγ^loxp^/^loxp^ mice on a C57/BL6 background were also used. Epor^loxp^/^loxp^/LysMCre^-/-^ or PPAR^loxp^/^loxp^/LysMCre^-/-^ mice were referred to as EPOR-C and PPARγ-C, respectively. Additionally, Epor^loxp^/^loxp^/LysMCre^+/+^ or PPAR^loxp^/^loxp^/LysMCre^+/+^ mice were referred to as EPOR-cKO and PPARγ-cKO mice, respectively. All mice were housed and bred in the animal facility at the Army Medical University under specific pathogen-free conditions. Male mice approximately 8 to 10 weeks old were used throughout these experiments.

### Microbe-Induced Peritonitis and Treatment

*E. coli* cultured in LB broth was harvested at mid-log phase (OD_600_ ≈ 0.8; 5 × 10^9^ c.f.u./mL) and then washed twice in sterile PBS. The *E. coli* culture was subsequently adjusted to a concentration of 2× the indicated c.f.u. in PBS; thus, a 500 µL injection was equivalent to 1× indicated c.f.u. Peritonitis infections were induced *via* an intraperitoneal injection of 500 µL of 1× indicated c.f.u. of *E. coli* into the abdominal cavity of mice. For calculation of resolution indices: T_max_, the time point of maximum PMN infiltration; Ψ_max_, the maximum PMN numbers; T_50_, the time point when PMN numbers are reduced to a maximum of 50%; Ψ_50_, 50% of maximum PMN; R_i_ (resolution interval, T_50_ – T_max_), the time period when 50% PMN are lost from exudates.

For treatment, the mice received an intraperitoneal injection of recombinant human erythropoietin (12, 13) (rhEPO; 5000IU/kg body weight; Sunshine Pharmaceutical, Shenyang, China) or intragastric administration of rosiglitazone or GW9662 (Sigma-Aldrich, St Louis, MO, USA) at the indicated time. For treatment with antibiotics, ciprofloxacin (Sigma-Aldrich, St Louis, MO, USA) was given 2 hrs after the *E. coli* injection. In addition, in some experiments, mice were injected intraperitoneally with anti-CD36 antibodies (ab17044 [clone FA6-152]; Abcam, Cambridge, UK) or mouse IgG1 (ab81032 [clone NCG01]; Abcam, Cambridge, UK).

At the time of sampling, body temperature was measured using an infrared thermometer. Mice were sacrificed, peritoneal exudate was collected with 1 mL PBS, and bacterial counts were determined using a coating plate. The total cell count was measured using a hemocytometer *via* light microscopy. Exudate cells were pelleted for flow cytometry. Furthermore, macrophages were isolated from single-cell suspensions using Anti-F4/80 MicroBeads UltraPure (130-110-443; Miltenyi Biotec GmbH, Bergisch Gladbach, Germany) according to the manufacturer’s instructions. Selected cells were subsequently used to extract total RNA for subsequent reverse transcription and real-time quantitative PCR detection. In some special experiments, lung tissues of mice with peritonitis were collected for H&E staining.

### Mouse Model of *S. aureus* Infection and Treatment

After the dorsal skin of the mouse was shaved, 2 mL of air was injected subcutaneously on days 0 and 3 to create a single pouch. *S. aureus* was grown overnight in BHI broth, washed twice in sterile PBS, and was subsequently adjusted to a concentration of 4 × 10^5^ c.f.u. in PBS, allowing for a 500 µL injection to contain 2 × 10^5^ c.f.u. On day 4, mice were given 500 µL of 2 × 10^5^ c.f.u. of *S. aureus* by intra-pouch injection. After 2 hrs, rhEPO or vancomycin hydrochloride (BBI Life Science, Shanghai, China) was injected into the dorsal skin pouch of mice. Mice were sacrificed 24 hrs after treatment; thereafter, skin pouch exudate was collected with 1 mL PBS, and bacterial counts were determined using a coating plate. The total cell count was measured using a hemocytometer *via* light microscopy. Exudate cells were pelleted for flow cytometry, and skin tissues of *S. aureus*-infected mice were collected for H&E staining.

### Flow Cytometry

Single-cell suspensions from cultured cells were harvested from mice *via* peritoneal lavage or skin pouch lavage; the cells were then washed and counted before being reacted with anti-CD16/32 antibodies (101302 [clone 93]; BioLegend, San Diego, CA, USA) to block the Fc receptors. The following antibodies were used: F4/80 (123116[clone BM8]; BioLegend, San Diego, CA, USA), Ly6G (M100LB-02E[clone HM36]; Sungene Biotech, Beijing, China) EPOR (MBS769048[polyclonal]; Mybiosource, San Diego, CA, USA), anti-rabbit IgG (406421[Poly4064]; BioLegend, San Diego, CA, USA), p-Jak2 (Ab219728[E132]; Abcam, Cambridge, UK), annexin V (640918; BioLegend, San Diego, CA, USA), *E. coli* antibody (GTX40856[polyclonal]; GeneTex, Irvine, CA, USA), PPAR-γ (PA5-25757[polyclonal] Invitrogen, California, USA), and CD36 (102606[clone HM36]; BioLegend, San Diego, CA, USA). In some specific experiments, cells were fixed and permeabilized with the Cytofix/Cytoperm Fixation/Permeabilization Solution Kit (BD Biosciences, Franklin Lakes, NJ, USA). Cell surface and intracellular staining were performed in accordance to the manufacturers’ instructions. Data were collected with a BD FACSCanto II analyzer (Becton Dickinson, Franklin Lakes, NJ, USA) and analysed with FlowJo software (Tree Star, Ashland, OR, USA).

Supernatants collected from cultured cells, peritoneal lavage, or skin pouch lavage were used for FACS analysis of inflammatory cytokines. The concentrations of MCP-1, IL-6, TNF-α, IL-β, IFN-γ, and IL-10 were determined using a mouse inflammation panel (BioLegend, San Diego, CA, USA) according to the manufacturer’s instructions.

### Experiments *In Vitro*

Mice were injected with 3% thioglycolic acid (Sigma-Aldrich, St Louis, MO, USA) and 72 hrs later, the abdominal cavity was washed with 5 mL sterile PBS twice to collect the cells of the peritoneal fluid. The collected cells were seeded in a 10 cm^2^ low adhesion dish (Corning, NY, USA) in DMEM with 10% FCS (Gibco, Grand Island, NY, USA), and cultured at 37°C and 5% CO2 overnight. The non-adherent cells were removed, leaving purified macrophages. In selected experiments, cells were pre-treated with the following reagents: rhEPO (20 IU/mL), 10 µM rosiglitazone, 1 µg/mL anti-CD36 antibody (clone FA6-152), 1 µg/mL mouse IgG1 (clone NCG01), or 10 µM GW9662. For some experiments, heat-inactivated *E. coli* was added to purified macrophages (10 c.f.u. per macrophage) prior to RNA isolation.

In *in vitro* phagocytosis experiments, bacteria were stained with 5 µL BacLight green bacterial stain (Molecular Probes, Invitrogen, California, USA) for 15 min at 37 °C. Macrophages were incubated with fluorescent-labeled *E. coli* at 10:1 ratio (*E. coli*: macrophages) for 30 min at 4°C (negative control) or 37°C. Cold PBS was added to terminate the reaction and cells were collected by centrifugation. Flow cytometry staining and analyses were performed as previously described.

### Bacterial Killing Assay

A suspension (1 × 10^7^ cells/mL) of purified peritoneal macrophages was prepared. Additionally, a bacterial suspension was prepared and contained approximately 2 × 10^8^ c.f.u./mL. Then, 100 μL of the cell suspension and 50 μL of the bacterial suspension were incubated for 45 min at 37°C. For control measurements, 100 μL sterile PBS and 50 μL bacterial suspension were incubated. The cells were recovered by centrifugation for 5 min at 400 ×*g*. The supernatant was reasonably diluted, and the pellets were resuspended in 0.1% Triton-X100 solution and reasonably diluted, from which 50 µL of the dilution was plated onto an LB-agar plate and was incubated for 24 h. Cell colonies were counted using the following equation:

Killing number = number of colonies in the control supernatant – number of colonies in the experimental supernatant– colony number of pellets.

### Immunofluorescence Assay

*E. coli* cells were collected in mid-log phase at OD_600_ ≈ 0.8, washed with sterile PBS, and adjusted to 3 × 10^9^ c.f.u./mL. CFSE (Selleck Chemicals, Shanghai, China) was added to the cells for 15 min resulting in a final concentration of 40 µM. This solution was incubated at 37°C for 15 min, in the dark, while gently rocking or shaking to ensure even staining. Bacteria collected by centrifugation were washed with sterile PBS. To heat-kill bacteria, the cells were incubated at 65°C for 15 min and stored at 4°C for later use.

A total of 3 × 10^5^ purified macrophages were labeled with the membrane dye PKH26 according to the manufacturer’s instructions (Sigma-Aldrich, St Louis, MO, USA), and seeded on glass slides in a 24-well plate to enable attachment. Cells were pre-treated with rhEPO or PBS for 24 hrs and then exposed to the bacteria (3 × 10^6^ c.f.u.) for 30 min. After phagocytosis, extracellular bacteria were removed by washing with sterile PBS. Cells were fixed with 2% paraformaldehyde, and nuclei were stained with DAPI (Biyuntian, Shanghai, China). The glass slides were removed from the 24-well plate, sealed with anti-fluorescence quenching sealing tablets, and placed on the slide. The findings were scanned under the Olympus system microscopes BX51 (Olympus, Tokyo, Japan) at 400× magnification. Identical exposure conditions and images were employed for all preparations using DP Manager software (Olympus, Tokyo, Japan).

### ELISA

Peritoneal fluid was used to detect the concentrations of EPO (R&D Systems, Minneapolis, MN, USA) using an ELISA kit according to the manufacturer’s instructions.

### Real-Time Quantitative PCR

RNA was isolated from cells sorted by magnetic beads or cultures of purified macrophages using the RNA fast 200 Kit (Fastagen, Shanghai, China) according to the manufacturer’s instructions. Reverse transcription was performed using a reverse transcription kit (Takara, Tokyo, Japan). qPCR was performed using SYBR Green qPCR Master Mix (MedChemExpress, Monmouth Junction, NJ, USA). qPCR was run on the CFX96 detection system (Bio-Rad Laboratories, Hemel Hempstead, UK); gene expression for each sample was normalized to β-actin for the mouse reference gene, and the differences were determined using the 2△C(t) calculation. Primers with the following sequences were used: CD36 forward 5′-ATGGGCTGTGATCGGAACTG-3′, reverse 5′-TTTGCCACGTCATCTGGGTTT-3′; PPARγ forward 5′-TCGCTGATGCACTGCCTATG-3′, reverse 5′-GAGAGGTCCACAGAGCTGATT-3′; EPOR forward 5′-GGTGAGTCACGAAAGTCATGT-3′, reverse 5′-CGGCACAAAACTCGATGTGTC-3′; β-actin forward 5′-GGCTGTATTCCCCTCCATCG-3′, reverse 5′-CCAGTTGGTAACAATGCCATGT-3′.

### Histological Assessment

Skin and lung samples were harvested, fixed in 4% paraformaldehyde, dehydrated, bisected, mounted in paraffin, and sectioned for H&E and periodic acid-Schiff staining according to the manufacturer’s protocol (Sigma-Aldrich, St Louis, MO, USA).

Lung injury scores were quantified by an investigator blinded to the treatment groups using the following criteria. Lung injury was assessed on a scale of 0–2 for each of the following criteria: i) neutrophils in the alveolar space, ii) neutrophils in the interstitial space, iii) number of hyaline membranes, iv) amount of proteinaceous debris, and v) extent of alveolar septal thickening. The final injury score was derived from the following calculation: Score = [20 × (i) + 14 × (ii) + 7 × (iii) + 7 × (iv) + 2 × (v)]/(number of fields × 100).

### Statistics

All values in the figures and text are expressed as mean ± SEM. Significance was calculated using one-way ANOVA with Tukey’s post hoc test for multiple comparisons or Student’s t-test for two groups meeting the normal distribution criteria. Survival rate was analysed *via* the log-rank test. For all statistical analyses, the statistical significance was represented by a single asterisk (P < 0. 05), two asterisks (P < 0. 01), or three asterisks (P < 0. 001), or four asterisks (P < 0. 0001) using GraphPad Prism 6.0 (https://www.graphpad.com/scientific-software/prism/).

## Results

### Macrophage EPO Signaling Is Temporally Activated During Self-Limited *E. coli*-Initiated Infections

Given that *E. coli* infections are an important worldwide health concern (21), we investigated the role of EPO in an *E. coli*-initiated infection resolution. We first detected the temporal expression of EPO and EPOR in a self-limited infection model induced by intraperitoneal administration of *E. coli* (10^5^ c.f.u. per mouse) (22). To quantitatively evaluate the infection resolution, the commonly used resolution indices were applied. Resolution indices are defined as the time point when polymorphonuclear neutrophil (PMN) numbers reach their maximum values (T_max_), the time point when PMN numbers are reduced to a 50% maximum (T_50_), and resolution interval (R_i_), the time interval in which the PMN numbers are reduced from the maximum values to 50% (22–24).

**Figure 1:**
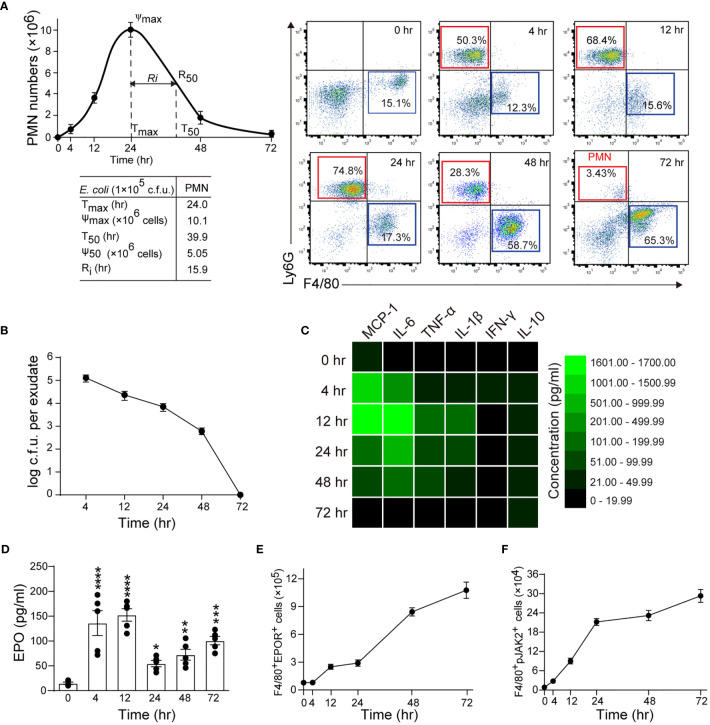
Macrophage EPO signaling is temporally activated in self-limited *E. coli*-initiated infections. Mice were inoculated with *E*. *coli* at 1 × 10^5^ c.f.u. by intraperitoneal injection (n = 5 for each time point). **(A)**: Left panel: time course of peritoneal PMN numbers and resolution indices (n = 5). Right panel: representative flow cytometric dot plots of peritoneal leukocytes. **(B)**: Time course of bacterial titers in peritoneal lavage fluids (n = 5). **(C)**: Heatmap of peritoneal inflammatory cytokines (n = 3), see histogram in **Figure S1C**. **(D–F)**: Time course of EPO concentrations in peritoneal lavage fluids **(D)**, peritoneal exudates of F4/80^+^EPOR^+^ macrophages **(E),** and peritoneal exudates of F4/80^+^pJak2^+^ macrophages **(F)** (n = 5). Data are representative of at least two independent experiments. Results were expressed as mean ± SEM. *P < 0.05, **P < 0.01, ***P < 0.001 and ****P < 0.0001 compared to 0 hrs. Statistics: One-way ANOVA with Tukey’s post hoc test for multiple comparisons **(D)**.

In this model, the peritoneal PMN count increased rapidly, peaked at about 24 hrs, followed by a gradual decrease to background levels at around 72 hrs, with a R_i_ of approximately 15.9 hrs (**Figure 1A**). The macrophage counts increased steadily from 4 to 48 hrs and then decreased (**Figures 1A** and **S1A**). Moreover, bacteria count in peritoneal lavages were completely cleared in 72 hrs (**Figures 1B** and **S1B**). We further observed that the proinflammatory cytokines MCP-1, IL-6, TNF-α, IL-1β, and IFN-γ rapidly increased and then quickly decreased to baseline levels at around 72 hrs (**Figures 1C** and **S1C**). While the anti-inflammatory cytokine IL-10 was also quickly induced, it gradually decreased and remained high at 72 hrs (**Figure 1C** and **S1C**). Therefore, the timely suppression of PMNs, invading bacteria and proinflammatory cytokines, together with the induction of antiphlogistic macrophages (such as efferocytosis) and anti-inflammatory cytokines indicate a self-resolving infection.

In this self-limited infection model, the EPO concentration in the peritoneal fluid at all observed time points (4, 12, 24, 48, and 72 hrs) was significantly higher than at baseline and peaked 4 and 12 hrs following infection (**Figure 1D**). Consequently, we detected EPOR levels in exudate leukocytes. Anti-EPOR antibody bound to normal macrophages but not to EPOR knockout (KO) macrophages, confirming its high specificity to EPOR (**Figures S1D, E**). Following *E. coli* infection, EPOR was mainly detected on macrophages but not PMNs, in line with our previous observations (12) (**Figure S1F**). The accumulation of EPOR^+^ macrophages in the abdominal cavity continuously increased (**Figure 1E**). Furthermore, we observed an increased macrophage EPO signaling activation, as indicated by phosphorylated JAK2 (p-JAK2), which is the sole direct signaling molecule downstream of EPOR in exudate leukocytes (**Figure 1F**). Collectively, these results suggest that a correlation exists between macrophage EPO signaling activation and infection resolution.

### Macrophage EPO Signaling Regulates Resolution of *E. coli*-Initiated Infections

As EPOR was mainly detected in macrophages during the self-limited *E. coli* peritonitis, we next sought to investigate the role of macrophage EPO signaling in infection resolution. We generated the myeloid *EPOR^−/−^* (EPOR-cKO) mice by crossing *LysM-Cre^+/+^* mice with *EPOR^loxp/loxp^* mice (13). In 10-week-old *EPOR-cKO* mice, the composition of macrophages and PMNs in spleens, lymph nodes (13), and the peritoneal cavity was similar to that of WT mice (**Figures S2A, B**). *LysM-Cre^+/+^/EPOR^+/+^* (EPOR-C) mice were used as controls.

**Figure 2:**
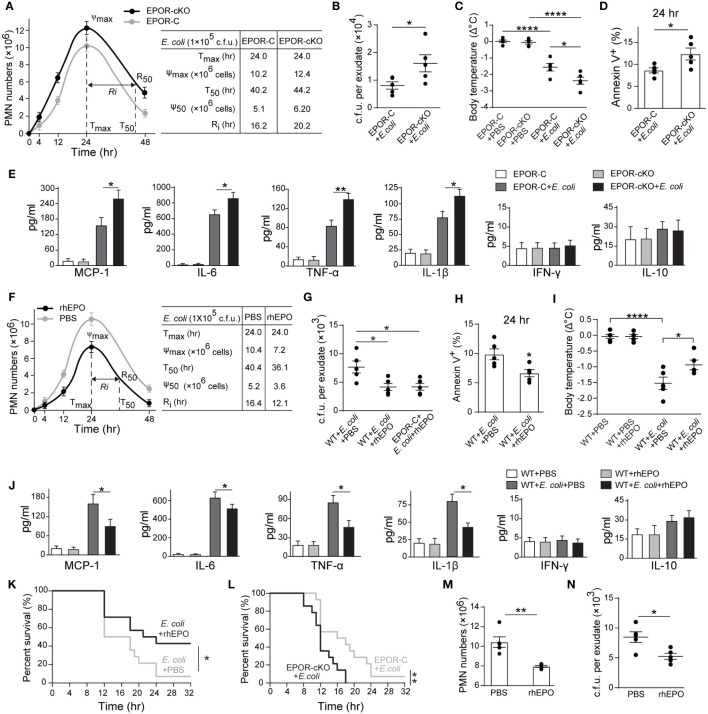
Macrophage EPO signaling regulates resolution of *E. coli*-initiated infections. For **(A–E)**, EPO-C, and EPOR-cKO mice were inoculated with 1 × 10^5^ c.f.u. *E. coli* (n = 5 for each group at each time point), **(A)** Time course of peritoneal PMN numbers and resolution indices (n = 5). **(B)**: Bacterial titers of peritoneal lavage fluids at 24 hrs (n = 5). **(C)** Changes in body temperatures expressed as mean of Δ°C (temperature at 24 h–temperature at 0 h, n = 5). **(D)** Percentage of apoptotic peritoneal leukocytes at 24 h (n = 5). **(E)** Inflammatory cytokines in peritoneal lavage fluids at 24 h (n = 3). For **(F–J)**, WT mice were inoculated with 1 × 10^5^ c.f.u. *E. coli* (n = 5 for each group at each time point) together with rhEPO (5,000 IU/kg) or PBS. **(F)** Time course of peritoneal PMN numbers and resolution indices (n = 5). **(G)** Bacterial titers of peritoneal lavage fluids at 24 hrs (n = 5). **(H)** Percentage of apoptotic peritoneal leukocytes at 24 hrs (n = 5). **(I)** Changes in body temperatures of (n = 5). **(J)** Inflammatory cytokines in peritoneal lavage fluids at 24 hrs (n = 3). **(K)** Percent survival of *E. coli* (5 × 10^7^ c.f.u.) inoculated WT mice alone or with 5,000 IU/kg of rhEPO (n = 14 for each group). **(L)** Percent survival of *E. coli* (5 × 10^7^ c.f.u.) inoculated EPO-C and EPOR-cKO mice (n = 14 for each group). For M-N, WT mice were inoculated with 1 × 10^5^ c.f.u. *E. coli*, 12 hours after infection, mice were treated with rhEPO (5,000 IU/kg) or PBS (n = 5 for each group), and 12 hours after rhEPO treatment, mice were sacrificed for measurement. **(M)** Peritoneal PMN numbers (n = 5). **(N)** Bacterial titers of peritoneal lavage fluids (n = 5). Data are representative of at least two independent experiments. Results were expressed as mean ± SEM. *P < 0.05, **P < 0.01, and ****P < 0.0001. Statistics: unpaired two-tailed Student’s t-test **(B, D, H, M, N)**, one-way ANOVA with Tukey’s post hoc test for multiple comparisons **(C, E, G, I, J)** or Log-Rank test **(K, L)**.

In the 10^5^ c.f.u. *E. coli* -induced peritonitis, higher exudate PMN infiltration was observed in the EPOR-cKO group compared to the control group (**Figures 2A** and **S2C**). Furthermore, myeloid EPOR deficiency resulted in a prolonged R_i_ (approximately 20 hrs in the EPOR-cKO mice and approximately 16 hrs in the control mice) (**Figure 2A**), indicating the important role of macrophage EPO signaling in promoting infection resolution. Accordingly, the bacteria count in peritoneal lavages was significantly higher in the EPOR-cKO mice compared with the control mice at 24 hrs (**Figure 2B**). Infected mice developed hypothermia with 1.58 ± 0.27°C decrements in body temperature at 24 hrs. Myeloid EPOR deficiency further exacerbated this body temperature decrease (**Figure 2C**). Furthermore, the apoptotic cell count in exudates was significantly increased by myeloid EPOR deficiency, indicating delayed resolution (**Figures 2D** and **S2D**). The levels of the MCP-1, IL-6, TNF-α, and IL-1β in exudates were higher in EPOR-cKO mice compared to control mice (**Figure 2E**). These data suggest that macrophage EPO signaling is essential for infection resolution.

We further investigated whether recombinant human EPO (rhEPO) promotes infection resolution in the 10^5^ c.f.u. *E. coli* -induced peritonitis in WT mice. rhEPO administrated together with *E. coli* resulted in significant decreases in exudate PMN infiltration (from 10.4 × 10^6^ to 7.2 × 10^6^ at 24 hrs), shortened the Ri (from 16 to 12 hrs) (**Figure 2F**), and reduced peritoneal exudate bacteria counts (**Figure 2G**) and the peritoneal exudate apoptotic cell counts (**Figures 2H** and **S2E**). Moreover, rhEPO further reduced the body temperature (**Figure 2I**) and the levels of MCP-1, IL-6, TNF-α, and IL-1β in the exudates compared to the control (**Figures 2J** and **S2F**). However, administration of rhEPO to the EPOR-cKO mice following infection did not result in a significant decrease in the R_i_ (**Figure S2G** **)**, indicating the central role of macrophage EPO signaling to infection resolution. Moreover, the lethal dose of *E. coli* (5 × 10^7^ c.f.u.) resulted in only 5% survival in WT mice and rhEPO significantly increased survival to about 40% (**Figure 2K**). Furthermore, the lethal dose of *E. coli* induced more death in the EPOR-cKO mice than in the control mice (**Figure 2L**). In addition, rhEPO administration at the peak of inflammation (12 h after E. coli injection) also caused a decrease in the neutrophil and bacteria counts at 24** h** (**Figures 2M, N**).

Collectively, these data demonstrate that macrophage EPO signaling is essential for infection resolution and exogenous EPO promotes acute infection resolution.

### EPO Enhances Noninflammatory Clearance of *E. coli* by Macrophages *In Vitro* and *In Vivo*

**Figure 3:**
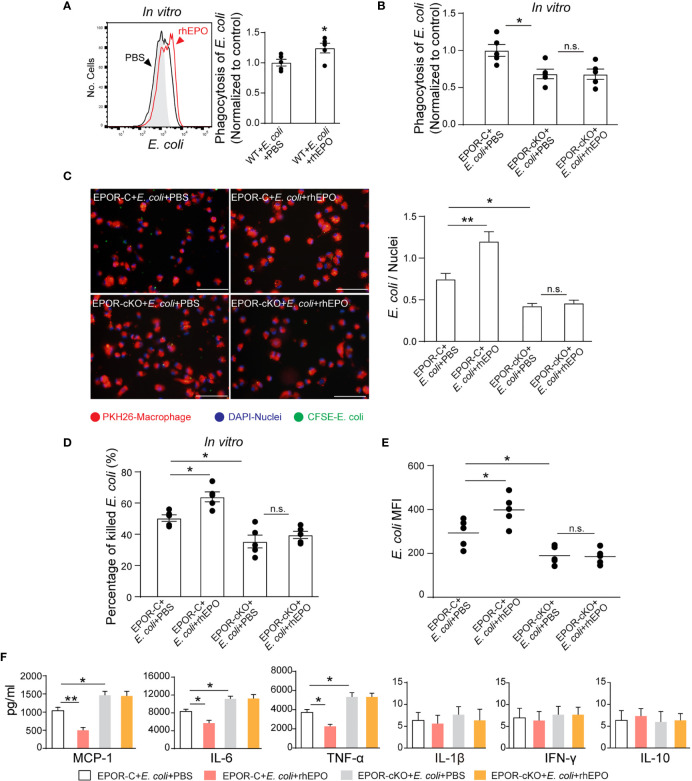
EPO enhances macrophage clearance of *E. coli in vitro* and *in vivo*. **(A)**: *In vitro* cultured WT mice thioglycolate-elicited peritoneal macrophages were pretreated with rhEPO (20 IU/ml) or PBS for 24 hrs, cells were then collected and incubated with fluorescently labeled *E. coli* for 30 min (macrophage: *E. coli* = 1: 10) at 37°C or 4°C (Gray, non-phagocytosis control), phagocytosis was analyzed by flow cytometry (n = 5). Statistical graph depicts the increase in MFI; phagocytosis by PBS-treated cells was normalized and used as a control. **(B)**: Thioglycolate-elicited peritoneal macrophages from EPOR-C or EPOR-cKO mice were pretreated with rhEPO (20 IU/ml) or PBS for 24 hrs. Cells were then collected and incubated with fluorescently labeled *E. coli* for 30 min (macrophage: *E. coli* = 1: 10), phagocytosis of *E. coli* was assayed by flow cytometry (n = 5). Phagocytosis by EPOR-C cells was normalized to control. **(C)**: Thioglycolate-elicited peritoneal macrophages from EPOR-C or EPOR-cKO mice were treated with rhEPO (20 IU/ml) or PBS for 24 hrs and then seeded with fluorescently labeled *E. coli* (macrophage: *E. coli* = 1: 10) for 30 min, uptake of *E. coli* was detected by microscopy (Bars, 50 μM). Statistical graph represents quantification of engulfed *E. coli* (n = 5). **(D)**: Thioglycolate-elicited peritoneal macrophages from EPOR-C or EPOR-cKO mice were cultured with rhEPO (20 IU/ml) or PBS for 24 hrs and then incubated with *E. coli* (macrophage: *E. coli* = 1: 10) for indicated times. *In vitro* bacterial killing activities of macrophages were performed (n = 5) as described in Materials and Methods. **(E)**: EPOR-C and EPOR-cKO mice were intraperitoneally inoculated with *E. coli* at 1 × 10^5^ c.f.u. together with rhEPO (5,000 IU/kg) or PBS by intraperitoneal injection. After 24 hrs, exudate leukocytes were lavaged and intracellular *E. coli* levels in macrophages were analyzed by flow cytometry (n = 5). **(F)**: *In vitro* cultured EPOR-C and EPOR-cKO mice thioglycolate-elicited peritoneal macrophages were stimulated with rhEPO (20 IU/ml) or PBS plus heat deactivated *E. coli* (macrophage: *E. coli* = 1: 10) for 24 hrs, inflammatory cytokines in medium supernatant were assayed (n = 3). Data are representative of at least two independent experiments. Results were expressed as mean ± SEM. ns, not statistically significant. *P < 0.05, **P < 0.01. Statistics: unpaired two-tailed Student’s t-test **(A)** or one-way ANOVA with Tukey’s post hoc test for multiple comparisons **(B–F)**. MFI, mean fluorescence intensity.

Considering the central role of bacterial clearance in infection resolution and the restricted localization of EPOR in macrophages, we evaluated whether EPO influences macrophage containment of *E. coli in vitro*. To score ingestion, *E. coli* were labeled with the BacLight green bacterial stain for flow cytometry (**Figure S3A**). In some cases, fluorescence microscopy was used to confirm internalization (**Figure 3C**). rhEPO increased *E. coli* engulfment by approximately 37% (**Figures 3A, C**). Moreover, phagocytosis of *E. coli* was reduced by approximately 40% by EPOR deficient macrophages compared with normal macrophages (**Figures 3B, C**). Furthermore, the conventional plate count assay showed that rhEPO enhanced the macrophage killing of *E. coli* by approximately 30% and that the EPOR KO decreased the macrophage killing of *E. coli* by around 40% (**Figure 3D**). Moreover, rhEPO did not exhibit direct anti-bacterial activity (**Figure S3B**). Together, these results indicate that EPO promotes macrophage clearance of *E. coli in vitro*.

Consequently, we investigated EPOR-dependent bacteria suppression *in vivo*. The intracellular *E. coli* was measured with an *E. coli*-specific antibody by flow cytometry (**Figure S3C**). In the 10^5^ c.f.u. *E. coli* -induced peritonitis, the *E. coli* engulfment by F4/80^+^ macrophages was reduced by ~35% in EPOR-cKO mice compared with control mice (**Figure 3E**). Moreover, administration of rhEPO promoted the phagocytosis of *E. coli* by F4/80^+^ macrophages in WT mice by ~35% (**Figure 3E**).

Subsequently, we examined whether EPO modulates cytokine expression during macrophage phagocytosis of *E. coli in vitro*. In the presence of *E. coli*, rhEPO significantly reduced the concentrations of IL-6, TNF-α, and MCP-1 in supernatants. However, the EPOR deficiency significantly enhanced the levels of IL-6, TNF-α, and MCP-1 (**Figure 3F**). Collectively, these results demonstrate that EPO promotes the nonphlogistic macrophage clearance of *E. coli*.

### EPO Promotes Noninflammatory Macrophage Clearance of *E. coli* Through PPARγ

**Figure 4:**
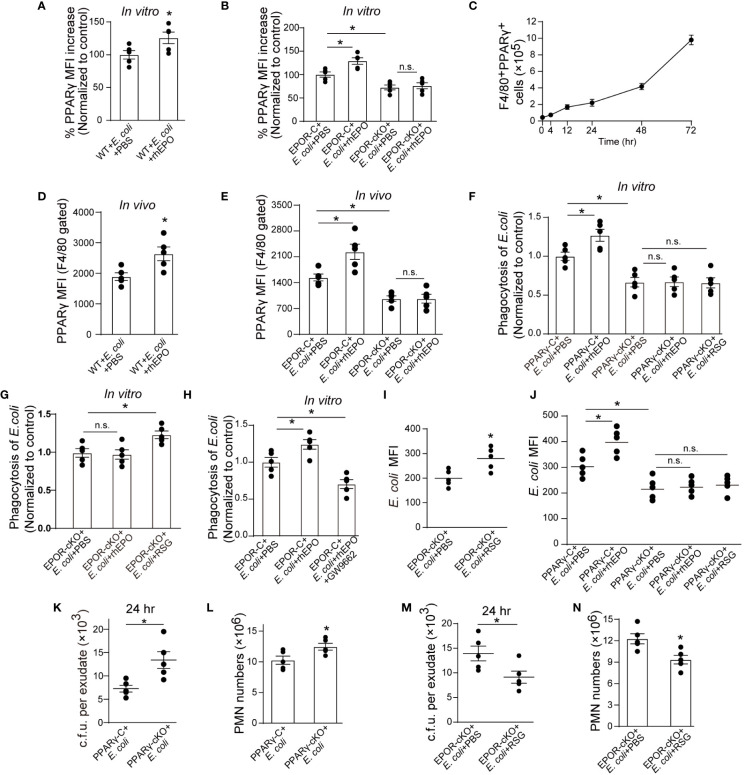
EPO promotes macrophage clearance of *E. coli* through PPARγ. **(A, B)**: Thioglycolate-elicited peritoneal macrophages from WT mice **(A)** or EPOR-cKO mice **(B)** were stimulated with rhEPO (20 IU/ml) or PBS in presence of heat deactivated *E. coli* (macrophage: *E. coli* = 1: 10) for 24 hrs and PPARγ expression was evaluated by flow cytometry (n = 5). **(C)**: WT mice were inoculated with *E. coli* at 1 × 10^5^ c.f.u. and peritoneal exudates of F4/80^+^PPARγ^+^ macrophages were assayed by flow cytometry at indicated time points (n = 5). **(D, E):** WT **(D)** or EPOR-cKO **(E)** mice were inoculated with *E. coli* at 1 × 10^5^ c.f.u. together with rhEPO (5,000 IU/kg) or PBS for 24 hrs and then peritoneal macrophage PPARγ levels were measured (n = 5). **(F–H)**: Thioglycolate-elicited peritoneal macrophages were cultured with rhEPO (20 IU/ml, 24 hrs), RSG (10 μM, 48 hrs), GW9662 (10 μM, 48 hrs) or PBS and then incubated with fluorescently labeled *E*. *coli* for 30 min (macrophage: *E. coli* = 1: 10) for phagocytosis assay (n = 5). **(I)**: EPOR-cKO mice were pretreated with RSG (10 mg/kg, i.g.) or PBS for 5 days. On day 6, mice were inoculated with *E. coli* at 1 × 10^5^ c.f.u. Intracellular *E. coli* levels in macrophages were analyzed at 24 hrs (n = 5). **(J)**: PPARγ-C and PPARγ-cKO mice were inoculated with *E. coli* at 10^5^ c.f.u. together with rhEPO (5,000 IU/kg), RSG (pretreated by 10 mg/kg, i.g. for 5 days) or PBS for 24 hrs. Intracellular *E. coli* levels in macrophages were analyzed at 24 hrs (n = 5). **(K**, **L)**: PPARγ-C and PPARγ-cKO mice were inoculated with *E. coli* at 1 × 10^5^ c.f.u. (n = 5). **(M, N)**: EPOR-cKO mice were pretreated with RSG (10 mg/kg, i.g.) or PBS for 5 days. On day 6, mice were inoculated with *E. coli* at 1 × 10^5^ c.f.u. (n = 5). *E. coli* exudates in peritoneal lavage fluids **(K, M)**, peritoneal exudates PMN cells **(L, N)** were assayed at 24 hrs. Data are representative of at least two independent experiments. Results were expressed as mean ± SEM. ns, not statistically significant. *P < 0.05. Statistics: unpaired two-tailed Student’s t-test **(A, D, I, K–N)** or one-way ANOVA with Tukey’s post hoc test for multiple comparisons **(B, E–H, J)**.

Consequently, we investigated mechanisms underlying EPOR-mediated *E. coli* clearance. We have previously reported that under homeostatic conditions, EPO promotes efferocytosis through PPARγ (13), which regulates the expression of various phagocyte engulfment receptors (25). Thus, we sought to explore whether PPARγ also mediates EPO-enhanced *E. coli* clearance. The myeloid *Pparg^−/−^* (PPARγ-cKO) mice were self-generated by crossing *LysM-Cre^+/+^* mice with *Pparg^loxp/loxp^* mice (26) We first detected whether EPO modulated macrophage PPARγ expression in the presence of *E. coli*. rhEPO increased the mRNA and protein levels of macrophage PPARγ (**Figures 4A, B** and **S4A, B**) and genetic deletion of *Epor* reduced PPARγ *in vitro* (**Figures 4B** and **S4B**). In the 1 × 10^5^ c.f.u. *E. coli*-induced peritonitis, the accumulation of PPARγ^+^ macrophages in the abdominal cavity continuously increased (**Figure 4C**), consistent with the accumulation of EPOR^+^ macrophages (**Figure 1E**). Moreover, the administration of rhEPO significantly enhanced levels of macrophage PPARγ (**Figures 4D, E** and **S4C, D**) and myeloid EPOR deficiency strongly reduced levels of macrophage PPARγ during *E. coli*-induced peritonitis (**Figures 4E** and **S4D**). Therefore, EPO upregulated macrophage PPARγ expression during *E. coli* phagocytosis *in vitro* and *in vivo.*

Subsequently, we investigated whether EPO enhances macrophage engulfment of *E. coli via* PPARγ *in vitro*. Genetic deletion of macrophage *Pparg* results in decreased uptake of *E. coli* and rhEPO did not increase *E. coli* phagocytosis in PPARγ deficient macrophages (**Figure 4F**). However, rosiglitazone (RSG, a PPARγ agonist) significantly enhanced *E. coli* engulfment in EPOR deficient macrophages (**Figure 4G**). Moreover, GW9662 (a PPARγ antagonist) abolished rhEPO inducing *E. coli* phagocytosis in WT macrophages (**Figure 4H**), indicating that EPO functions through PPARγ to increase *E. coli* engulfment. Similar results were observed *in vivo* when rhEPO or RSG were administrated to EPOR-cKO, PPARγ-cKO, or WT mice (**Figures 4I, J**).

There is increasing evidence demonstrating the important role of PPARγ in suppressing macrophage inflammatory cytokine expression (25, 27). We also observed that the PPARγ antagonist GW9662 abolished rhEPO reduction in levels of IL-6, TNF-α, and MCP-1 in macrophages (**Figure S4E**) *in vitro*. In EPOR-deficient macrophages, RSG but not rhEPO significantly reduced levels of IL-6, TNF-α, and MCP-1 in the presence of *E. coli* (**Figure S4F**). However, neither RSG nor rhEPO decreased concentrations of IL-6, TNF-α, and MCP-1 in PPARγ deficient macrophages (**Figure S4G**).

We further investigated the effects of macrophage PPARγ on infection resolution in *E. coli*-initiated peritonitis. In PPARγ-cKO mice, the exudate *E. coli* counts (**Figure 4K**), numbers of exudate PMNs (**Figure 4L**), and concentrations of exudate IL-6, TNF-α, IL-1β, and MCP-1 (**Figure S4H**) were significantly higher than those of the control mice for the 10^5^ c.f.u. *E. coli* -induced peritonitis. Moreover, in the EPOR-cKO mice, RSG treatment significantly reduced the exudate *E. coli* counts (**Figure 4M**), numbers of exudate PMNs (**Figure 4N**), and concentrations of exudate inflammatory cytokines (**Figure S4I**). In addition, GW9662 treatment in the WT mice suppressed rhEPO, reducing exudate E. coli counts (**Figure S4J**), exudate PMN numbers (**Figure S4K**), and concentrations of exudate inflammatory cytokines (**Figure S4L**). Collectively, these results indicate that macrophage EPO signaling enhances noninflammatory *E. coli* clearance through PPARγ to promote infection resolution.

### PPARγ-Induced CD36 Contributes to EPO-Enhanced Macrophage Phagocytosis of *E. coli*

PPARγ modulates the expression of a plethora of engulfment receptors to regulate macrophage phagocytosis (28, 29). CD36 is a well-known pivotal target of PPARγ-mediated gene expression and has been indicated as an important receptor for bacterial phagocytosis (30, 31). Therefore, we further investigated the contributions of CD36 in EPO increased bacterial clearance.

**Figure 5:**
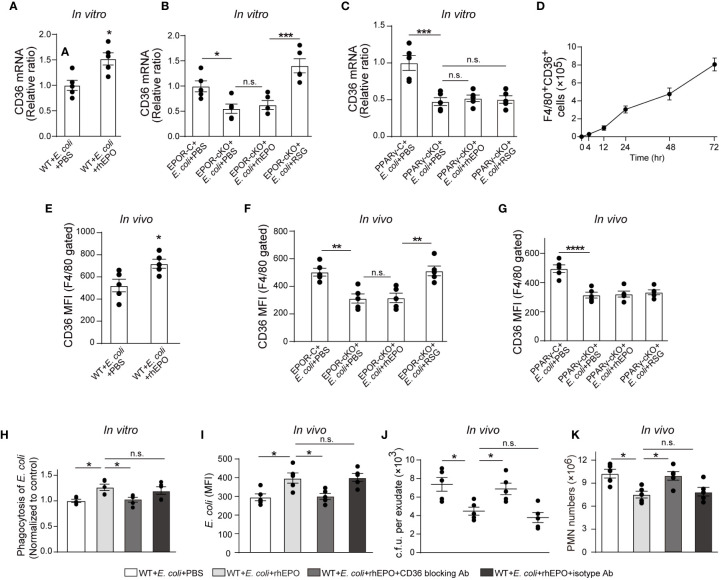
PPARγ-induced CD36 contributes to EPO-enhanced macrophage phagocytosis of *E. coli*. **(A)**: Thioglycolate-elicited peritoneal macrophages were incubated with rhEPO (20 IU/ml) or PBS in the presence of heat deactivated *E. coli* (macrophage: *E. coli* = 1: 10) for 24 hrs. CD36 transcript levels in purified macrophages were assayed by RT-qPCR (n = 5). **(B, C)**: Thioglycolate-elicited peritoneal macrophages from EPOR-cKO mice **(B)** or PPARγ-cKO mice **(C)** were incubated with rhEPO (20 IU/ml, 24 hrs), RSG (10 μM, 48 hrs) or PBS in the presence of heat deactivated *E. coli* (macrophage: *E. coli* = 1: 10, 24 hrs) and then macrophage expression of CD36 mRNA was detected (n = 5). **(D)**: WT mice were inoculated with *B coli* at 1 × 10^5^ c.f.u., F4/80^+^CD36^+^ peritoneal macrophages were assayed by flow cytometry (n = 5). **(E–G)**: WT mice **(E)**, EPOR-cKO mice **(F)** or PPARγ-cKO mice **(G)** were inoculated with *E. coli* at 10^5^ c.f.u. together with rhEPO (5,000 IU/kg), RSG (pretreated by 10 mg/kg, i.g. for 5 days) or PBS for 24 hrs and then peritoneal macrophages CD36 expression were assayed by flow cytometry (n = 5). **(H)**: Thioglycolate-elicited peritoneal macrophages from WT mice were treated with rhEPO (20 IU/ml), anti-CD36 blocking antibody (1μg per million cells), isotype-control antibody (1μg per million cells) or PBS for 24 hrs and then cells were collected and incubated with fluorescently labeled *E. coli* (macrophage: *E. coli* = 1: 10) for phagocytosis assay (n = 5). **(I–K)**: WT mice were inoculated with *E. coli* at 10^5^ c.f.u. together with rhEPO (5,000 IU/kg), anti-CD36 blocking antibody (3.3 mg/kg), isotype-control antibody (3.3 mg/kg) or PBS for 24 hrs. Intracellular *E. coli* levels in macrophages **(I)**, bacterial titers in peritoneal lavage fluids **(J)**, numbers of peritoneal PMN cells **(K)** were measured (n = 5). Data are representative of at least two independent experiments. Results were expressed as mean ± SEM. ns, not statistically significant. *P < 0.05, **P < 0.01, ***P < 0.001, ****P < 0.0001. Statistics: unpaired two-tailed Student’s t-test **(A, E)** or one-way ANOVA with Tukey’s post hoc test for multiple comparisons **(B, C, F–K)**.

In line with PPARγ, rhEPO induced the macrophage expression of CD36 (**Figure 5A** and **S5A**) in the presence of *E. coli in vitro*. Genetic deletion of *Epor* reduced levels of CD36 in macrophages (**Figures 5B** and **S5B**). Moreover, RSG but not rhEPO increased CD36 levels in EPOR KO macrophages (**Figure 5B** and **S5B**) and neither RSG nor rhEPO enhanced CD36 expression in PPARγ KO macrophages *in vitro* (**Figure 5C**), suggesting that EPO induced CD36 through PPARγ *in vitro*. In the 10^5^ c.f.u. *E. coli*-induced peritonitis, the accumulation of CD36^+^ macrophages in the abdominal cavity continuously increased (**Figure 5D**), suggesting the infiltration of EPOR^+^ macrophages (**Figure 1E**) and PPARγ^+^ macrophages (**Figure 4C**). Moreover, rhEPO increased macrophage CD36 expression in WT mice during *E. coli*-induced peritonitis (**Figure 5E**). However, CD36 levels in macrophages were significantly decreased in EPOR-cKO mice during peritonitis, which were restored by PPARγ agonist RSG, but not rhEPO (**Figure 5F**). In addition, macrophage CD36 levels were significantly reduced in PPARγ-cKO mice with or without rhEPO treatment during peritonitis (**Figure 5G**). These data indicate that EPO regulates macrophage CD36 expression *via* PPARγ during *E. coli*-induced peritonitis.

In view of these results, we investigated whether CD36 contributed to EPO-enhanced macrophage phagocytosis of *E. coli*. The CD36-specific blocking antibody abolished EPO-increased *E. coli* engulfment by macrophages *in vitro* (**Figure 5H**). In the *in vivo* study, CD36-specific blocking antibodies or the isotype control antibodies were administered together with *E. coli* to WT mice. Similarly to *in vitro* observations, the CD36-specific blocking antibody also diminished EPO-increased *E. coli* phagocytosis by macrophages (**Figure 5I**). Correspondingly, the EPO-reduced exudate *E. coli* counts were restored to Phosphate-buffered saline (PBS control levels by CD36-specific antibodies but not by isotype control antibodies during *E. coli* peritonitis (**Figure 5J**). Similar results were observed for exudate PMN counts (**Figures 5K** and **S5C**) and exudate cytokine levels (**Figure S5D**), indicating a role of CD36 in promoting *E. coli* phagocytosis and infection resolution.

Collectively, these observations suggest that EPO-induced macrophage CD36 enhances *E. coli* phagocytosis and promotes infection resolution.

### EPO Promotes Bacterial Clearance and Enhances Ciprofloxacin Actions in Resolution-Delayed *E. coli*-Initiated Infections

**Figure 6:**
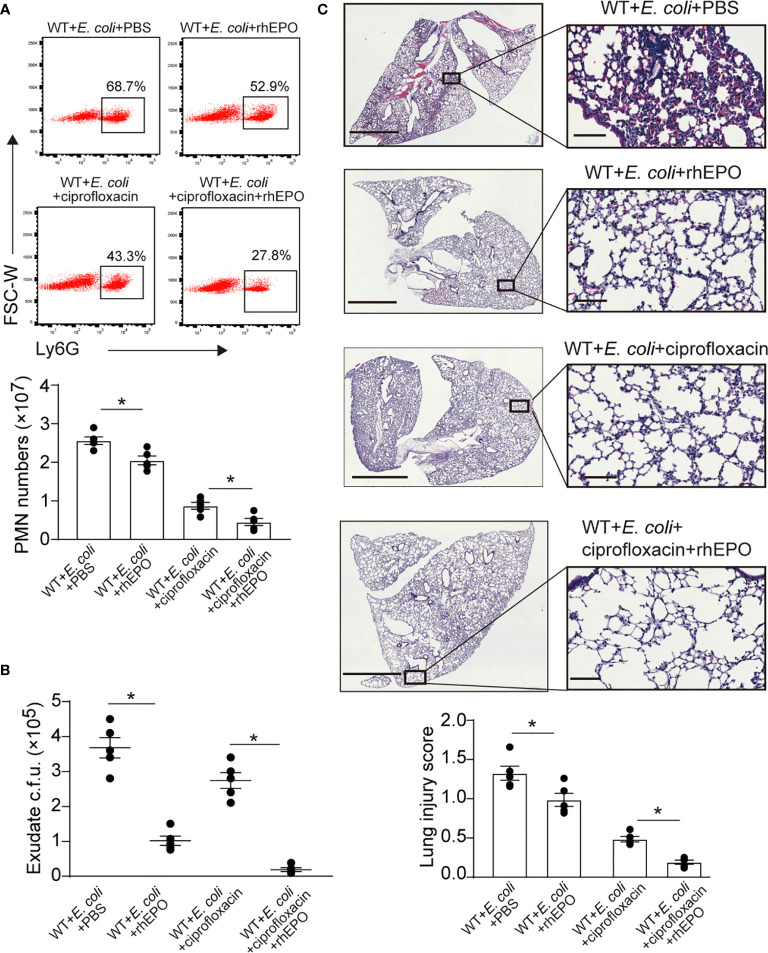
EPO promotes bacterial clearance and enhances ciprofloxacin actions in resolution-delayed *E. coli*-initiated infections. WT mice were inoculated with *E. coli* (5 × 10^6^ c.f.u.) by intraperitoneal injection. After 2 hrs, mice were treated with rhEPO (5,000 IU/kg), ciprofloxacin (12.5 mg/kg), ciprofloxacin (12.5 mg/kg) plus rhEPO (5,000 IU/kg) or PBS. After 24 hrs, mice were sacrificed (n = 5). **(A)**: Percentage and cell numbers of peritoneal PMN cells. **(B)**: Bacterial titers of peritoneal lavage fluids. **(C)**: Histopathology in lungs (2 mm for left bars,100 μM for right bars). The lung injury score was assessed as described in Materials and Methods. Data are representative of at least two independent experiments. Results were expressed as mean ± SEM. *P < 0.05. Statistics: one-way ANOVA with Tukey’s post hoc test for multiple comparisons **(A–C)**.

We next sought to investigate the effects of EPO on the delayed resolution of infection. A higher dose of *E. coli* (5 × 10^6^ c.f.u. per mouse) induced delayed resolution of bacterial peritonitis (22). rhEPO administrated together with *E. coli* significantly reduced exudate PMN infiltration (**Figure 6A**), exudate *E. coli* counts (**Figure 6B**), and levels of inflammatory cytokines IL-6, TNF-α, IL-1β, and MCP-1 (**Figure S6**) at 24 hrs. Moreover, pathological changes in the lung, particularly the infiltration of leukocytes, were significantly improved following rhEPO treatment compared to controls (**Figure 6C**).

We further demonstrated whether EPO-directed host response would improve antibiotic treatment. Sub-optimal doses of ciprofloxacin (22) alone significantly decreased exudate PMN infiltration (**Figure 6A**), exudate *E. coli* counts (**Figure 6B**), levels of IL-6, TNF-α, IL-1β, and MCP-1 (**Figure S6**), and leukocyte accumulation in the lungs (**Figure 6C**) at 24 hrs. rhEPO given together with ciprofloxacin further reduced exudate PMN numbers (**Figure 6A**), exudate E. coli counts (**Figure 6B**), leukocyte accumulation in the lungs (**Figure 6C**), and levels of IL-6, TNF-α, IL-1β, and MCP-1 (**Figure S6**) at 24 h compared to ciprofloxacin or rhEPO alone.

Collectively, these results indicate that EPO promotes bacterial clearance and improves ciprofloxacin actions in the delayed resolution of *E. coli*-initiated infections.

### EPO Promotes Bacterial Phagocytosis and Enhances Vancomycin Actions in *S. aureus*-Initiated Infections

While EPO enhanced the clearance of the Gram-negative pathogen *E. coli*, its effects on Gram-positive bacteria are unknown. *S. aureus* is a Gram-positive bacterial pathogen that is responsible for significant morbidity and mortality worldwide and it is the leading cause of skin infection (32–34). Therefore, we investigated the effects of EPO on *S. aureus* infection.

**Figure 7:**
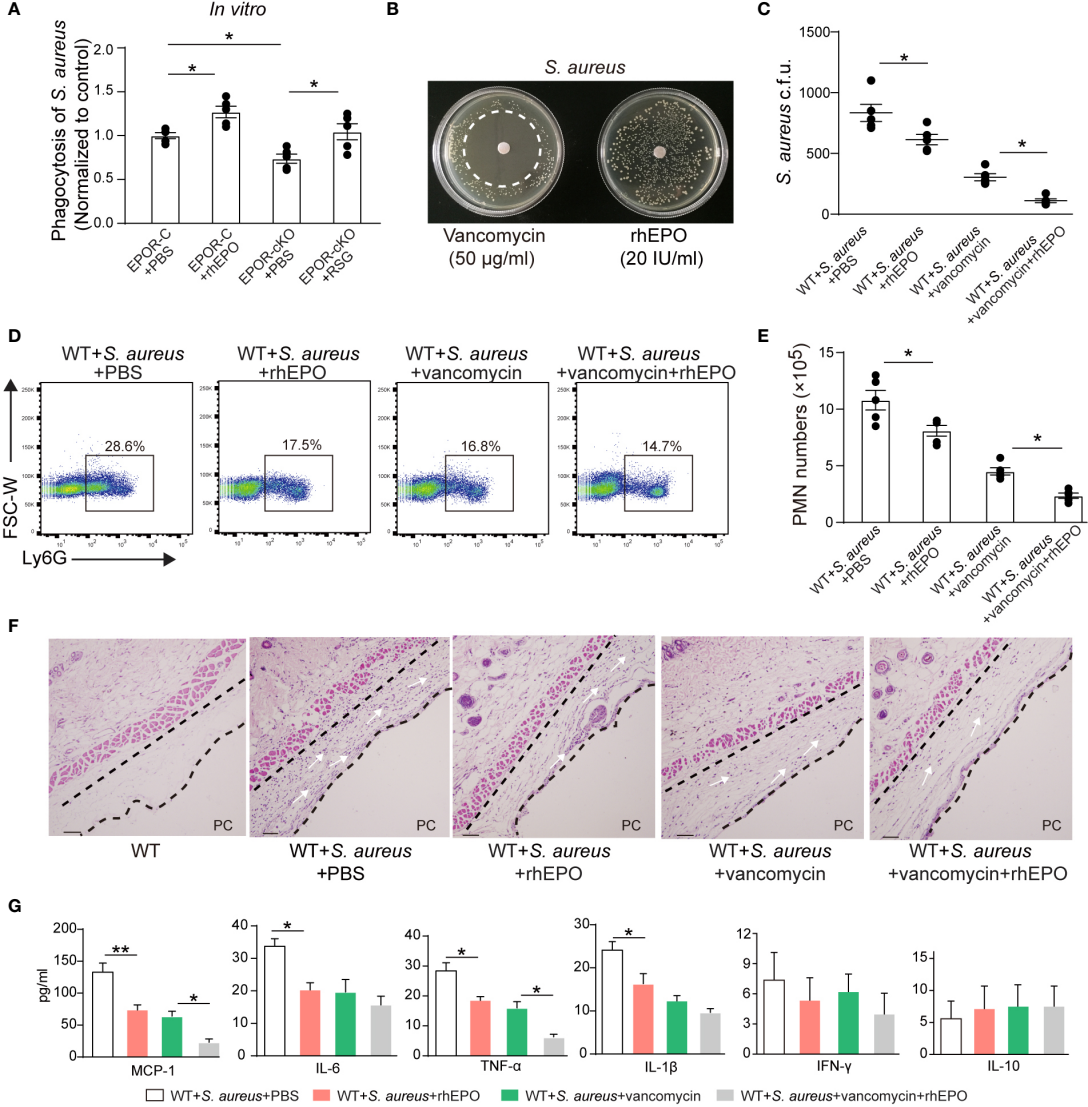
EPO promotes bacterial phagocytosis and enhances actions of vancomycin in S. *aureus*-initiated infections. **(A)**: Thioglycolate-elicited peritoneal macrophages from EPOR-C or EPOR-cKO mice were pretreated with rhEPO (20 IU/ml, 24 hrs), RSG (10 μM, 48 hrs) or PBS for 24 hrs and then incubated with fluorescently labeled S. aureus for 30 min (macrophage: S. aureus = 1: 10). In vitro phagocytosis of S. aureus by macrophages was analyzed by flow cytometry (n = 5). **(B)**: Vancomycin (50 μg/ml, as a positive control) or rhEPO (20 IU/ml) were each placed on BHI agar plates containing S. aureus (10^6^ c.f.u.). The zone of clearance was assessed after overnight incubation. Representative plates from each group are shown in photographs. **(C–G)**: WT mice dorsal pouches were given live S. aureus (2 × 10^5^ c.f.u.) together with rhEPO (5,000 IU/kg), vancomycin (50 mg/kg), both rhEPO (5,000 IU/kg) and vancomycin (50 mg/kg) or PBS by intra-pouch injection. Thereafter, pouch exudates were collected for the measurement. **(C)**: Bacterial counts of S. aureus (c.f.u.); **(D)**: Representative flow cytometric dot plots of neutrophils in pouch exudates; **(E)**: Exudate PMN numbers; **(F)**: skin HE staining (PC, pouch cavity. Bars, 50 μM) at 24 h (n = 5); **(G)**: Inflammatory cytokines in pouch exudates (n = 3). Data are representative of at least two independent experiments. Results were expressed as mean ± SEM. *P < 0.05, **P < 0.01. Statistics: one-way ANOVA with Tukey’s *post hoc* test for multiple comparisons **(B, C, E, G)**.

*In vitro*, rhEPO significantly enhanced macrophage containment of *S. aureus*. EPOR KO in macrophages significantly reduced *S. aureus* phagocytosis, which could be restored by the PPARγ agonist (**Figure 7A**). Moreover, rhEPO did not exhibit direct anti-bacterial activity in *S. aureus* (**Figure 7B**).

Thereafter, we determined the effect of EPO and vancomycin on *S. aureus*-initiated infections in murine dorsal skin pouches. rhEPO or sub-optimal doses of vancomycin alone significantly decreased exudate bacterial counts (**Figure 7C**), exudate PMN infiltration (**Figures 7D, E**), levels of IL-6, TNF-α, IL-1β, and MCP-1 (**Figure 7G**), and leukocyte infiltration within linings surrounding the pouch cavities (**Figure 7F**) at 24 hr. Treatment with both EPO and vancomycin further reduced exudate bacterial counts (**Figure 7C**), exudate PMN infiltration (**Figures 7D, E**), levels of inflammatory cytokines TNF-α and MCP-1 (**Figure 7G**), and leukocyte infiltration (**Figure 7F**) at 24 h compared to ciprofloxacin or rhEPO alone.

Given *S. aureus* rapidly acquires genes for drug resistance and the degree of antibiotic-resistant *S. aureus* has been greatly increased (33) we further investigated effects of EPO on methicillin-resistant *S. aureus* (MRSA) in murine dorsal skin pouches. *In vitro*, rhEPO significantly enhanced macrophage containment of MRSA. EPOR KO in macrophages significantly reduced MRSA engulfment, which could be restored by the PPARγ agonist (**Figure S7A**). Moreover, rhEPO did not exhibit direct anti-bacterial activity in MRSA *in vitro* (**Figure S7B**). rhEPO or sub-optimal doses of vancomycin alone significantly decreased exudate bacterial counts (**Figure S7C**), exudate PMN infiltration (**Figures S7D, E**), levels of IL-6, TNF-α, IL-1β, and MCP-1 (**Figure S7G**), and leukocyte infiltration within linings surrounding the pouch cavities (**Figure S7F**) at 24 hr. Treatment with both EPO and vancomycin further reduced exudate bacterial counts (**Figure S7C**), exudate PMN infiltration (**Figures S7D, E**), levels of inflammatory cytokines TNF-α and MCP-1 (**Figure S7G**), and leukocyte infiltration (**Figure S7F**) at 24 hr compared to ciprofloxacin or rhEPO alone. Together these results suggest that EPO promotes Gram-positive *S. aureus* phagocytosis and enhances vancomycin anti-bacterial actions in *S. aureus*-initiated infections.

## Discussion

Given the global rise of multidrug-resistant bacteria, novel mediators that promote infection resolution are of great interest. Here our results demonstrate that EPO is temporally induced during infections and EPO is anti-phlogistic, increases engulfment, promotes infection resolution, and enhances antibiotic action in *E. coli*- and *S. aureus*-initiated infections (**Figure S8**). While our previous investigations showed that EPO promoted apoptotic cell clearance the removal of foreign particles is different from apoptotic cell phagocytosis by recognition, uptake, and degradation of particles, leading to very different outcomes. Here we described for the first time that EPO enhanced bacterial phagocytosis and killing by macrophages which promoted infection resolution. Moreover, we report that the combination of EPO with antibiotics further increases the clearance of bacteria and improves the outcomes of infections, suggesting that combinational therapy with antibiotics and EPO may achieve a synergistic maximal benefit for treating infections.

Recent literature reports have revealed that infection/inflammation resolution is an active programmed response (7, 35, 36). During infections, pro-resolving mediators mainly function to promote macrophage clearance of pathogens, restitute barrier integrity, promote the repair of damaged tissues, and inhibit pain (7, 9, 35, 37). In this study, we found that EPO was induced following infection and promoted noninflammatory bacterial clearance. Moreover, extensive investigations have revealed that EPO is important in restoring tissue homeostasis following injury. The local upregulation of EPO and EPOR has been observed in a wide range of inflammatory responses or during injury (12, 15, 38–47. EPO exerted a direct cyto-protective effect on a variety of nonhematopoietic cells (14, 18, 48–50), protected intestinal epithelial barrier function (51), and alleviated neuropathic pain (52). Together with previous observations our results suggest that EPO is an endogenous pro-resolving molecule essential for infection resolution.

Furthermore, we demonstrate that PPARγ plays an important role in EPO-enhanced macrophage phagocytosis of *E. coli* and *S. aureus*. Consistent with our results, there is increasing evidence demonstrating that PPARγ plays an important role in inhibiting macrophage expression of inflammatory cytokines and mitigation of inflammation-induced host damage (25, 26, 28). Furthermore, PPARγ also enhanced the clearance of *Pseudomonas aeruginosa (P. aeruginosa)* (53) and the resolution of *S. aureus* skin infections (54) and *S. aureus* brain abscesses (55). In accordance with our observations, the PPARγ agonist ciglitazone enhanced *S. aureus* phagocytosis by microglia and attenuated the expression of inflammatory mediators in *S. aureus* brain abscesses (55). While existing research showed that PPARγ dependent induction of paraoxonase-2 drives *P. aeruginosa* clearance (53) and PPARγ dependent glucose and oxygen depletion augments the clearance of *S. aureus* (54), our results showed that PPARγ-induced CD36 contributes to EPO-enhanced macrophage phagocytosis of bacteria. CD36 is known to be induced following PPARγ activation in macrophages and has been implicated as an important receptor for bacterium phagocytosis, including *E. coli* and *S. aureus* (31). In accordance with our results, there is increasing evidence demonstrating that the activation of PPARγ enhances the clearance of *Plasmodium falciparum via* CD36 (30). Therefore, our data herein support the involvement of a macrophage PPARγ/CD36 pathway in host infection response.

In addition, we show that ameliorating the infection using rhEPO accelerated bacterial clearance and improved pathological outcome in *E. coli-* and *S. aureus-*initiated infections. However, in contrast to our observations, Nairz et al. found that rhEPO significantly reduced the survival of mice in a *Salmonella Typhimurium* sepsis mode by inhibiting pro-inflammatory immune effector pathways (56). This is most likely due to *Salmonella Typhimurium* being intracellular bacteria which have developed many sophisticated strategies to enter and survive within phagocytic cells. In contrast, both *E. coli* and *S. aureus* are extracellular bacteria that have evolved mechanisms to prevent their phagocytosis as part of their pathogenic profile (57, 58). Therefore, molecules that can enhance the bacterial phagocytosis may result in resistance to extracellular bacterial infections but be susceptible to intracellular bacterial infections. In line with these results, animals lacking PPARγ, identified here to work downstream of EPO to promote phagocytosis of bacteria, exhibited improved clearance of intracellular pathogens, including *Brucella abortis* (59), *Salmonella Typhimurium* (60), *Mycobacterium tuberculosis* (61), and *Listeria monocytogenes* (62). However, activation of PPARγ enhances the resolution of extracellular bacteria-induced infection, such as *P. aeruginosa* (53) and *S. aureus* (54, 55). Thus, the relative importance of EPO for defense against bacteria depends on pathogen biology and care must be taken when targeting EPO as a treatment for specific infectious diseases.

With the dramatic increase in antibiotic-resistant bacteria, host-directed therapeutics may assist in combating infectious diseases (4, 10). The present data show that EPO directly modulates host innate immunity enhancing phagocytosis, eliminating microbes, and thus accelerating resolution of infections. Moreover, we report that the combination of EPO with antibiotics further increases the clearance of bacteria and improves the outcomes of infections, suggesting that EPO may represent a potential adjunctive therapy with antibiotics for the treatment of certain bacterial infections. However, further studies will be needed to transfer our findings to human therapeutical settings.

## Data Availability Statement

The raw data supporting the conclusions of this article will be made available by the authors, without undue reservation.

## Ethics Statement

The animal study was reviewed and approved by Laboratory Animal Welfare and Ethics Committee of the Army Medical University.

## Author Contributions 

ZZ provided the idea and conceived and designed the experiments. FL and TL performed the experiments. WL, XZ, YL, JM, and CJ provided the technical support. BL and ZZ analyzed and interpreted the data. ZZ, FL, HG, and BL wrote the draft of the manuscript. ZZ revised the manuscript. ZZ, FZ, and BL supervised the study. All authors contributed to the article and approved the submitted version.

## Funding

This work was supported by a grant (2016YFA0502201) from the National Key Research Program of China (ZZ) and grants (81671559 and 32000638) from the National Natural Science Foundation of China (ZZ and BL).

## Conflict of Interest

The authors declare that the research was conducted in the absence of any commercial or financial relationships that could be constructed as a potential conflict of interest.
