# Supplementary material for: Erythropoietin Promotes Infection Resolution and Lowers Antibiotic Requirements in E. coli- and S. aureus-Initiated Infections
Source: Front Immunol. 2021 Apr 13;12:658715. doi: 10.3389/fimmu.2021.658715 (PMC8076604; doi:10.3389/fimmu.2021.658715)
Supplement: Supplementary file 1 [file Table_1.docx]

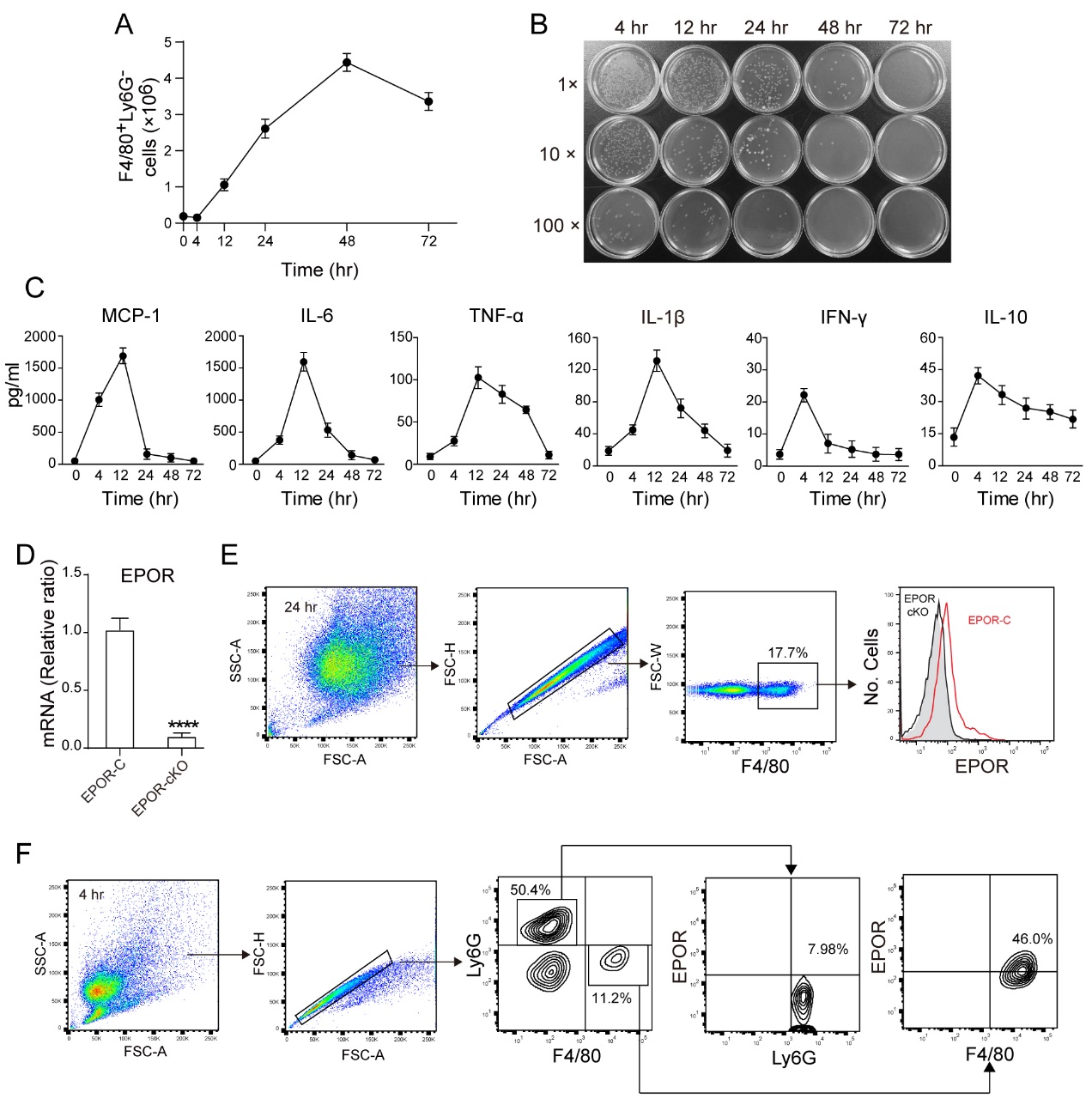


**Figure S1. Evaluation of self-limited *E. coli* infections in mice.**

For **A-C**, WT mice were inoculated with *E. coli* at 1 × 10^5^ c.f.u. by intraperitoneal injection (n = 5 for each time point). **a**: Time course of peritoneal F4/80+Ly6G- cells (n = 5). **B**: Representative photographs of LB agar plates with peritoneal lavage fluids (50 μl). **C**: Histogram of inflammatory cytokines in peritoneal lavage fluids (n = 3). **D**: *In vitro* cultured EPOR-C and EPOR-cKO mice thioglycolate-elicited peritoneal macrophages were collected, macrophage EPOR mRNA levels were assayed by RT-qPCR (n = 5). **E**: EPOR-C and EPOR-cKO mice were inoculated with *E. coli* at 1 × 10^5^ c.f.u. by intraperitoneal injection. Result depicts flow cytometric gating strategy for EPOR surface expression of peritoneal macrophages. Grey histogram is the fluorescence minus one (FMO) control. **F**: WT mice were inoculated with *E. coli* at 1 × 10^5^ c.f.u. by intraperitoneal injection. Result depicts flow cytometric analysis of EPOR distribution on peritoneal leukocytes. Data are representative of at least two independent experiments. Results were expressed as mean ± SEM. *****P* < 0.0001. Statistics: unpaired two-tailed Student’s t-test (D).


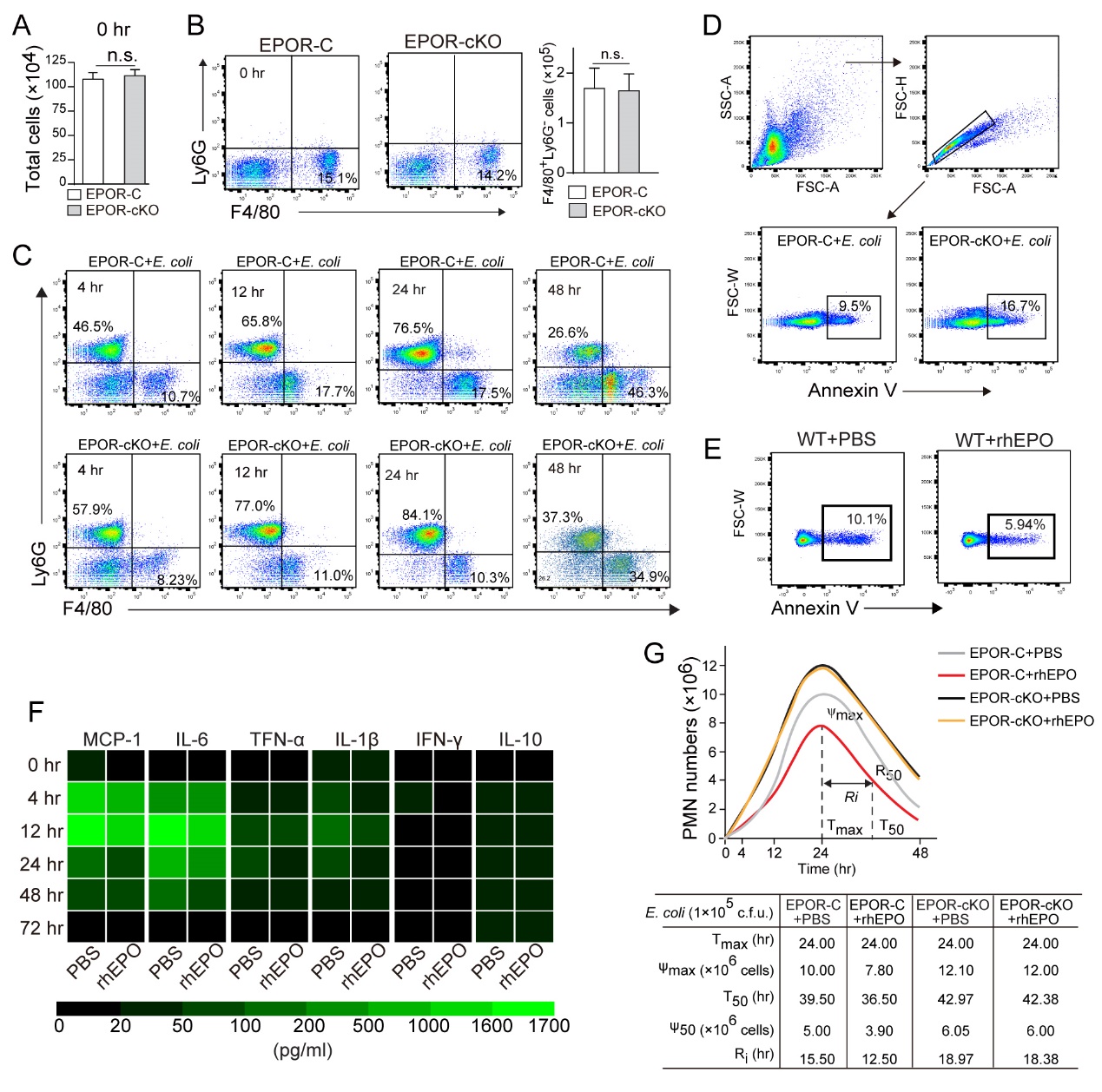


**Figure S2. Macrophage EPO signaling regulates resolution of *E. coli*-initiated self-limited infections.**

For **A-D**, EPOR-C and EPOR-cKO mice were inoculated with *E. coli* at 1 × 10^5^ c.f.u. by intraperitoneal injection, **A**: Total cell numbers in peritoneal lavage fluids at 0 hr (steady state, n = 5). **B**: Percentage and cell number of peritoneal F4/80+Ly6G- cells at 0 hr (steady state, n = 5). **C**: Representative flow cytometric dot plots of F4/80+Ly6G- cells and PMN of peritoneal exudate leukocytes in EPOR-C and EPOR-cKO mice. **D**: Representative flow cytometric dot plots of apoptotic peritoneal exudate leukocytes at 24 hr. For **E-F**, WT mice were inoculated with *E. coli* at 10^5^ c.f.u. by intraperitoneal injection, together with rhEPO (5,000 IU/kg) or PBS. **E**: Representative flow cytometric dot plots of apoptotic peritoneal exudate leukocytes at 24 hr. **F**: Heatmap of inflammatory cytokines in peritoneal lavage fluids. **G**: EPOR-C and EPOR-cKO mice were inoculated with *E. coli* (1 × 10^5^ c.f.u.) together with rhEPO (5,000 IU/kg) or PBS (n = 5 for each group at each time point). Left panel: Time course of peritoneal PMN numbers; Right panel: Resolution indices (n = 5). Data are representative of at least two independent experiments. Results were expressed as mean ± SEM. n.s.: not statistically significant. Statistics: unpaired two-tailed Student’s t-test (A, B).


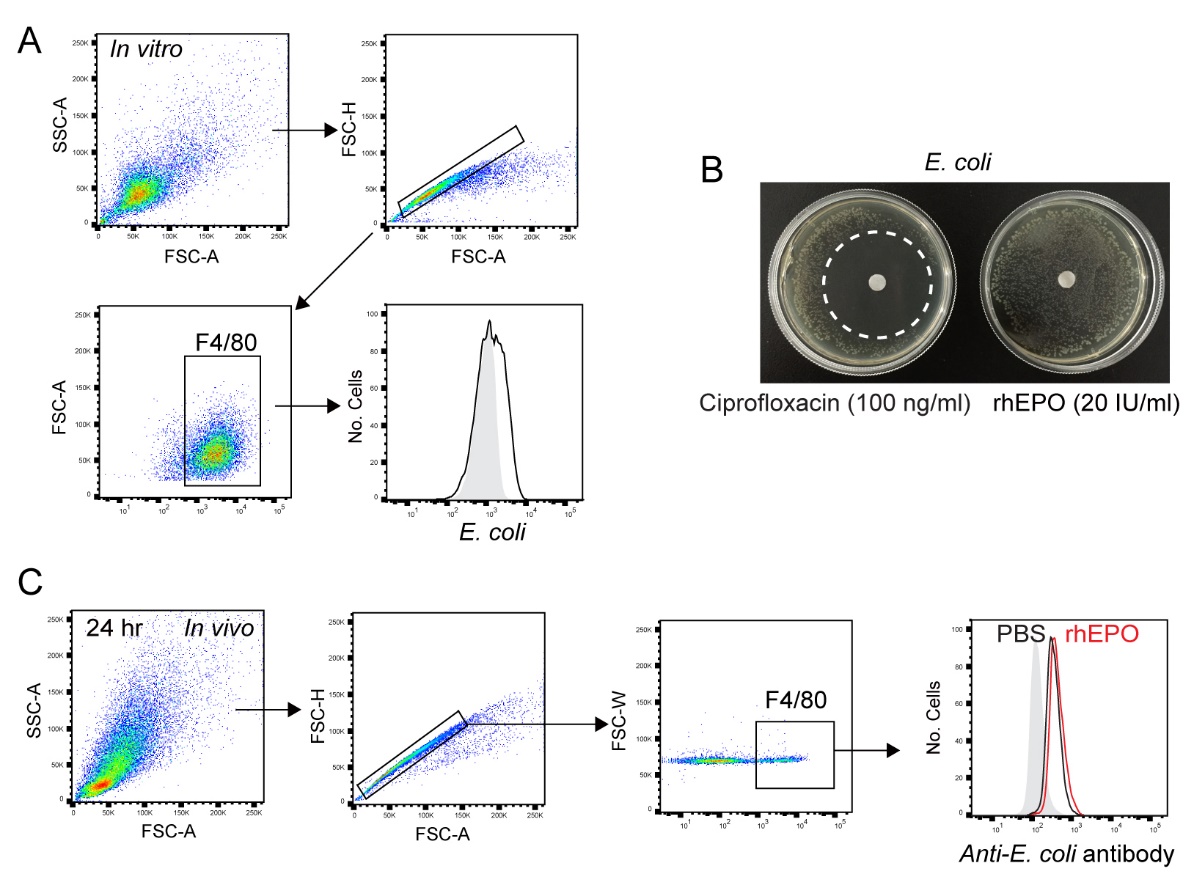


**Figure S3. EPO up-regulates macrophage clearance of *E. coli* *in vitro* and *in vivo*.**

**A**: *In vitro* cultured WT mice thioglycolate-elicited peritoneal macrophages were collected and incubated with fluorescently labeled *E. coli* for 30 min (macrophages : *E. coli* = 1 : 10), representative flow cytometric dot plots depict gating strategy for *in vitro* phagocytosis of *E. coli* by macrophages. **B**: Ciprofloxacin (100 ng/ml, as a positive control) or rhEPO (20 IU/ml) were each placed on LB agar plates containing *E. coli* (10^5^ c.f.u.). The zone of clearance was assessed after overnight incubation. Representatives from each group are shown in photographs. **C**: WT mice were inoculated with *E. coli* (10^5^ c.f.u.) together with rhEPO (5,000 IU/kg) or PBS, after 24 hrs, peritoneal leukocytes were collected, result depicts flow cytometric gating strategy for macrophage *in vivo* phagocytosis of *E. coli* using a *E. coli*-specific antibody. Grey histogram is the fluorescence minus one (FMO) control. Data are representative of at least two independent experiments.


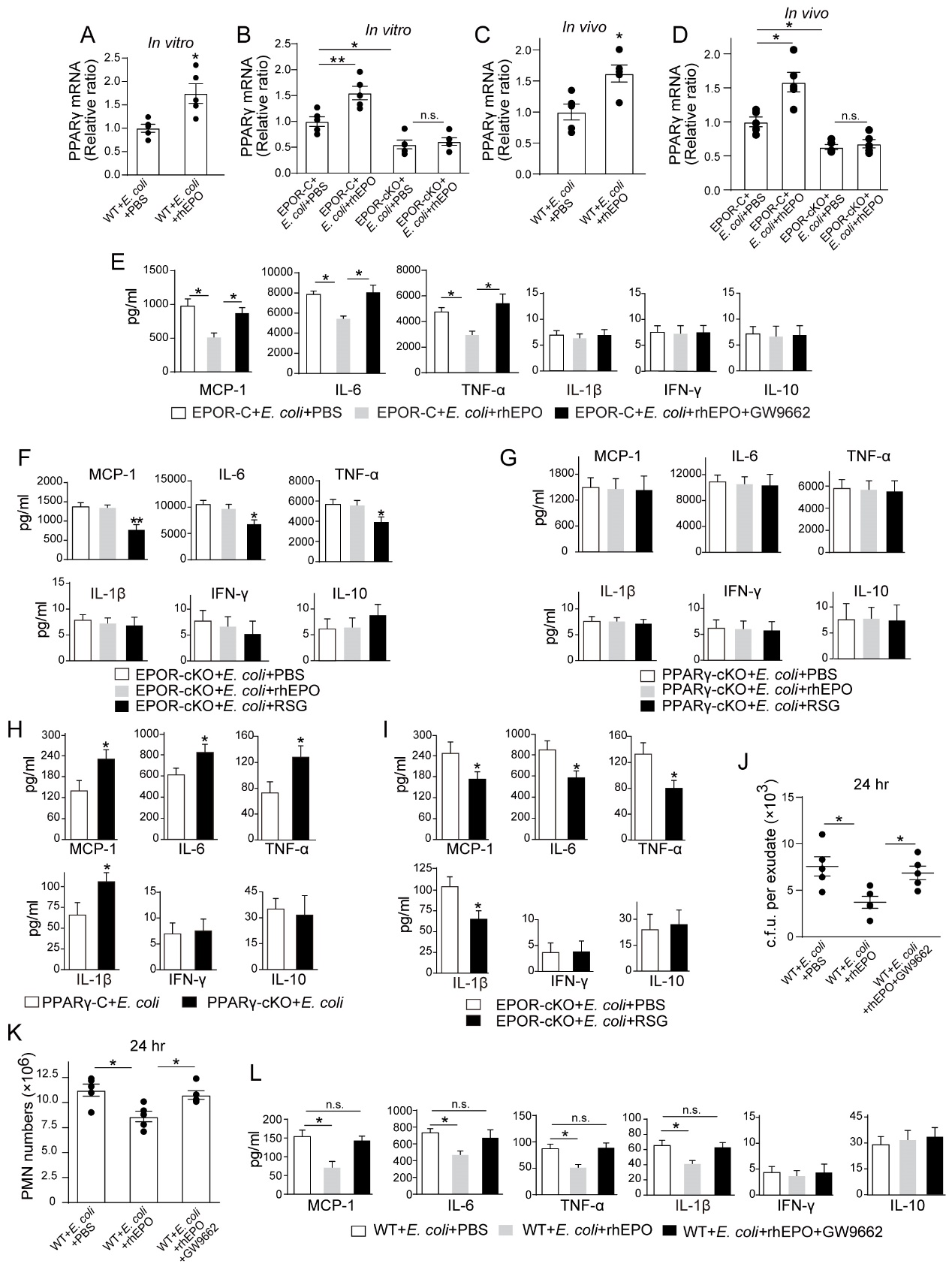


**Figure S4. EPO promotes macrophage clearance of *E. coli* through PPARγ.**

**A-B**: Thioglycolate-elicited peritoneal macrophages from WT mice (**A**) or EPOR-cKO mice (**B**) were stimulated with rhEPO (20 IU/ml) or PBS in presence of heat deactivated *E. coli* (macrophage: *E. coli* = 1: 10) for 24 hrs and PPARγ mRNA expression was evaluated by RT-qPCR (n = 5). **C**-**D**: WT (C) or EPOR-cKO (**D**) mice were inoculated with *E. coli* at 10^5^ c.f.u. together with rhEPO (5,000 IU/kg) or PBS for 24 hrs and then peritoneal macrophage PPARγ mRNA levels were measured by RT-qPCR (n = 5). **E**: Thioglycolate-elicited peritoneal macrophages from EPOR-C mice mice were cultured with rhEPO (20 IU/ml, 24 hrs), GW9662 (10 μM, 48 hrs) or PBS in presence of heat deactivated *E. coli* (macrophage: *E. coli* = 1: 10) for 24 hrs, inflammatory cytokines in the medium supernatant were assayed by flow cytometry (n = 3). **F**-G: Thioglycolate-elicited peritoneal macrophages from EPOR-cKO mice (**F**) or PPARγ-cKO mice (G) were cultured with rhEPO (20 IU/ml, 24 hrs), RSG (10 μM, 48 hrs) or PBS in presence of heat deactivated *E. coli* (macrophage: *E. coli* = 1: 10) for 24 hrs, inflammatory cytokines in the medium supernatant were assayed by flow cytometry (n = 3). **H**: PPARγ-C or PPARγ-cKO mice were inoculated with *E. coli* at 10^5^ c.f.u. by intraperitoneal injection, after 24 hrs, inflammatory cytokines in peritoneal lavage fluids were assayed at 24 hr by flow cytometry (n = 3). **I**: EPOR-cKO mice were pretreated with RSG (10 mg/kg, i.g.) or PBS for 5 days. On day 6, mice were inoculated with *E. coli* at 10^5^ c.f.u. and inflammatory cytokines in peritoneal lavage fluids were assayed at 24 hr (n = 3). **J-K:** WT mice were pretreated with rhEPO (5,000 IU/kg), both rhEPO (5,000 IU/kg) and GW9662 (10 mg/kg, i.g.) for 5 days or PBS for 24 hrs, then mice were inoculated with *E. coli* at 10^5^ c.f.u. (n = 5). *E. coli* exudates in peritoneal lavage fluids (**J**), peritoneal exudates PMN cells (**K**) and inflammatory cytokines in peritoneal lavage fluids (**L**, n = 3) were assayed at 24 hr. Data are representative of at least two independent experiments. Results were expressed as mean ± SEM. n.s.: not statistically significant. **P* < 0.05, ***P* < 0.01. Statistics: unpaired two-tailed Student’s t-test (A, C, H, I) or one-way ANOVA with Tukey’s post hoc test for multiple comparisons (B, D, E, F, G, J, K, L).


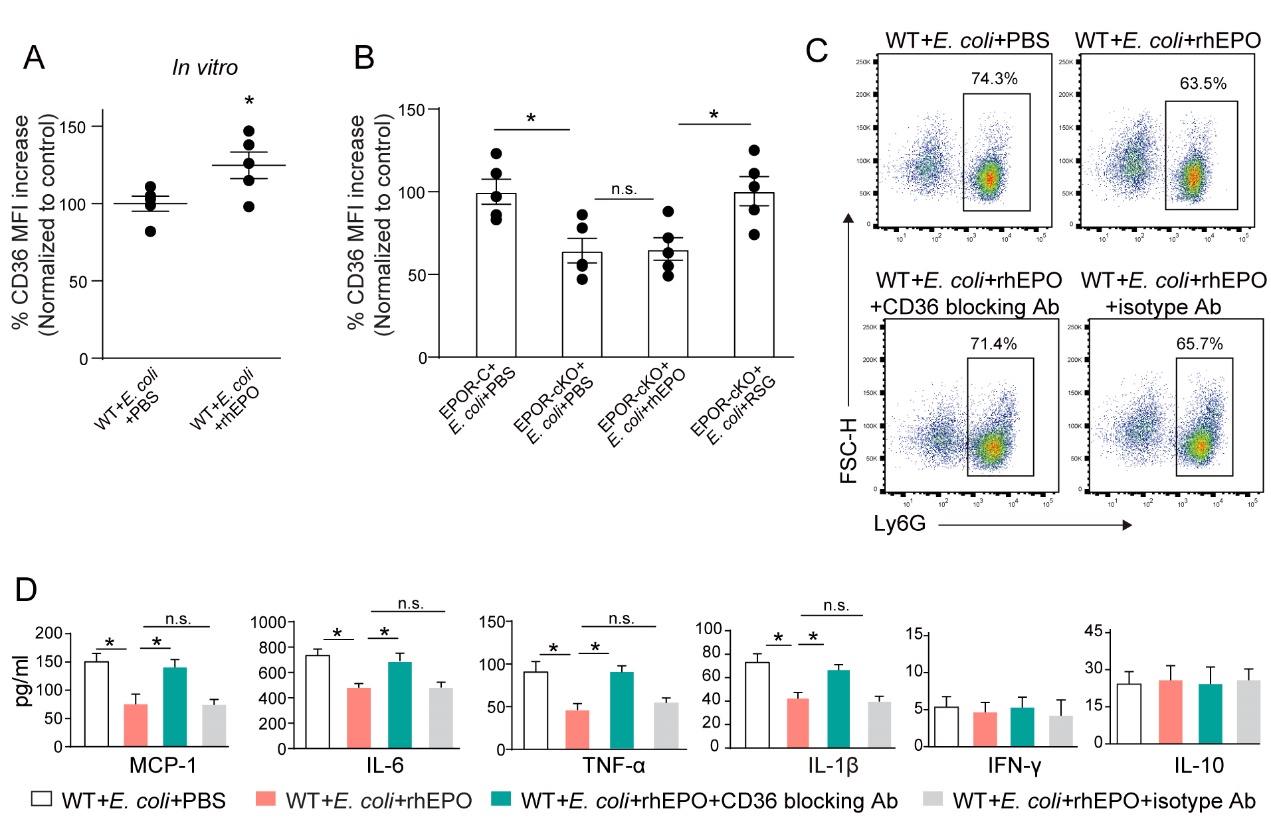


**Figure S5. PPARγ-induced CD36 contributes to EPO enhanced macrophage phagocytosis of *E. coli*.**

**A**: Thioglycolate-elicited peritoneal WT macrophages were incubated with rhEPO (20 IU/ml) or PBS in the presence of heat deactivated *E. coli* (macrophage: *E. coli* = 1: 10) for 24 hrs. The surface expression of macrophage CD36 was evaluated by flow cytometry (n = 5). **B**: Thioglycolate-elicited peritoneal macrophages from EPOR-cKO mice were incubated with rhEPO (20 IU/ml, 24 hrs), RSG (10 μM, 48 hrs) or PBS in the presence of heat deactivated *E. coli* (macrophage : *E. coli* = 1 : 10, 24 hrs) and then macrophage expression of CD36 was detected (n = 5). For **C-D**, WT mice were inoculated with *E. coli* at 1 × 10^5^ c.f.u. together with rhEPO (5,000 IU/kg), anti-CD36 blocking antibody (3.3 mg/kg), isotype-control antibody (3.3 mg/kg) or PBS for 24 hrs, numbers of peritoneal PMN cells **(C**) and inflammatory cytokines in peritoneal lavage fluids (**D**) were measured (n = 3). Data are representative of at least two independent experiments. Results were expressed as mean ± SEM. n.s.: not statistically significant. **P* < 0.05. Statistics: unpaired two-tailed Student’s t-test (A) or one-way ANOVA with Tukey’s post hoc test for multiple comparisons (B-D).


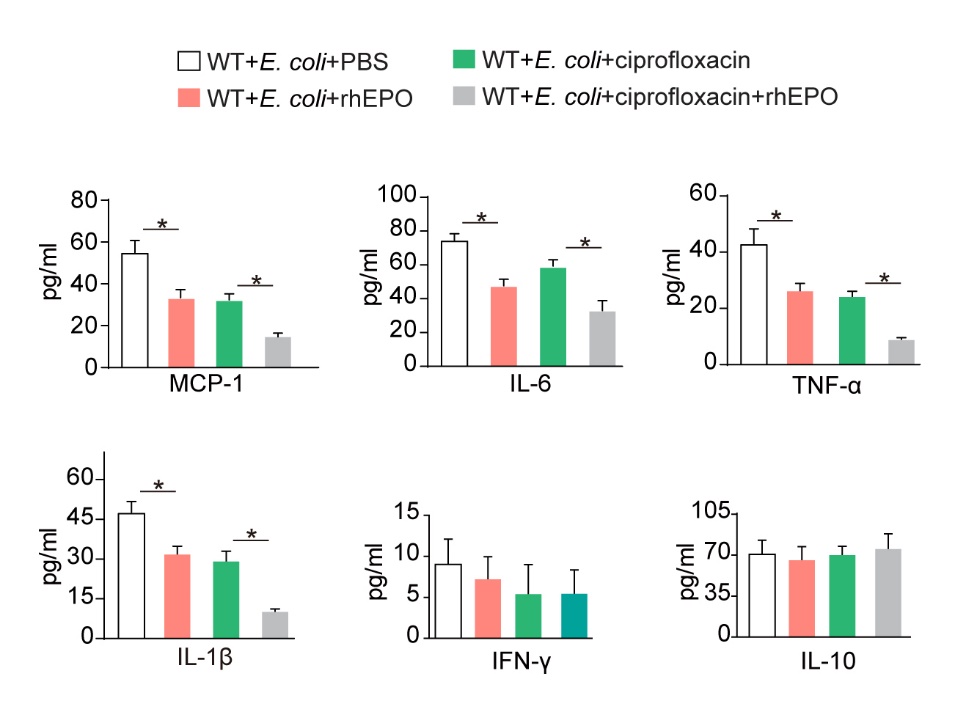


**Figure S6. EPO enhances actions of ciprofloxacin in resolution delayed *E. coli*-initiated infections.**

WT mice were inoculated with *E. coli* (5 × 10^6^ c.f.u.) by intraperitoneal injection together with rhEPO (5,000 IU/kg), ciprofloxacin (12.5 mg/kg), ciprofloxacin (12.5 mg/kg) plus rhEPO (5,000 IU/kg) or PBS. After 24 hrs, mice were sacrificed, and inflammatory cytokines of peritoneal lavage fluids were assayed (n = 3). Data are representative of at least two independent experiments. Results were expressed as mean ± SEM. **P* < 0.05. Statistics: one-way ANOVA with Tukey’s post hoc test for multiple comparisons.


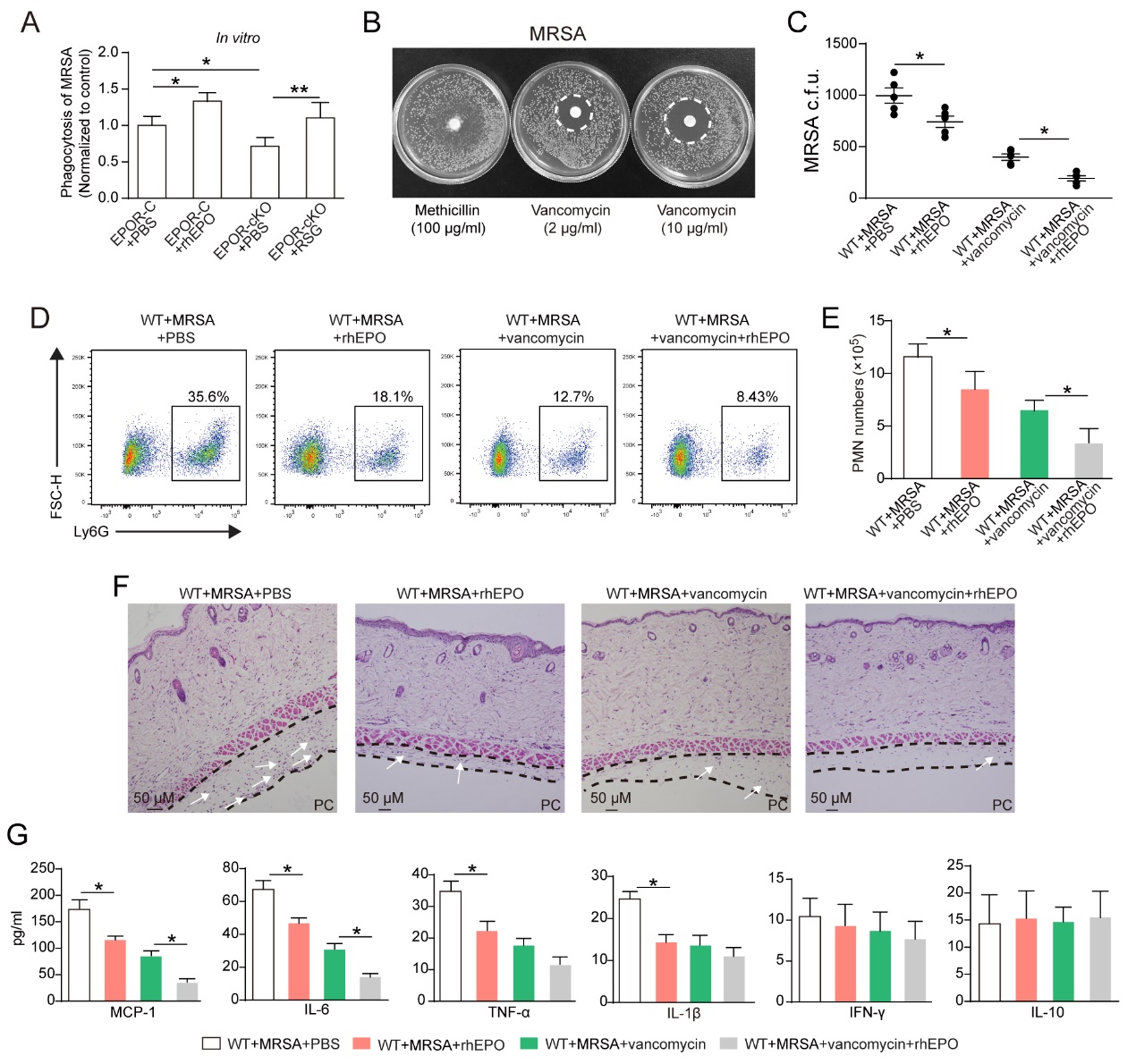


**Figure S7. EPO promotes bacterial phagocytosis and enhances actions of vancomycin in S. *aureus*-initiated infections.**

**A**: Thioglycolate-elicited peritoneal macrophages from EPOR-C or EPOR-cKO mice were pretreated with rhEPO (20 IU/ml, 24 hrs), RSG (10 μM, 48 hrs) or PBS for 24 hrs and then incubated with fluorescently labeled MRSA for 30 min (macrophage: MRSA = 1: 10). In vitro phagocytosis of MRSA by macrophages was analyzed by flow cytometry (n = 5). **B**: Vancomycin or methicillin were each placed on BHI agar plates containing MRSA (10^6^ c.f.u.). The zone of clearance was assessed after overnight incubation. Representative plates from each group are shown in photographs. **C-G**: WT mice dorsal pouches were given live MRSA (2 × 10^5^ c.f.u.) together with rhEPO (5,000 IU/kg), vancomycin (50 mg/kg), both rhEPO (5,000 IU/kg) and vancomycin (50 mg/kg) or PBS by intra-pouch injection. Thereafter, pouch exudates were collected for the measurement. **C**: Bacterial counts of MRSA (c.f.u.); **D**: Representative flow cytometric dot plots of PMN in pouch exudates; **E**: Exudate PMN numbers; **F**: skin HE staining (PC, pouch cavity. Bars, 50 μM) at 24 hr (n = 5); **G**: Inflammatory cytokines in pouch exudates (n = 3). Data are representative of at least two independent experiments. Results were expressed as mean ± SEM. **P* < 0.05, ***P* < 0.01. Statistics: one-way ANOVA with Tukey’s post hoc test for multiple comparisons (B, C, E, G).


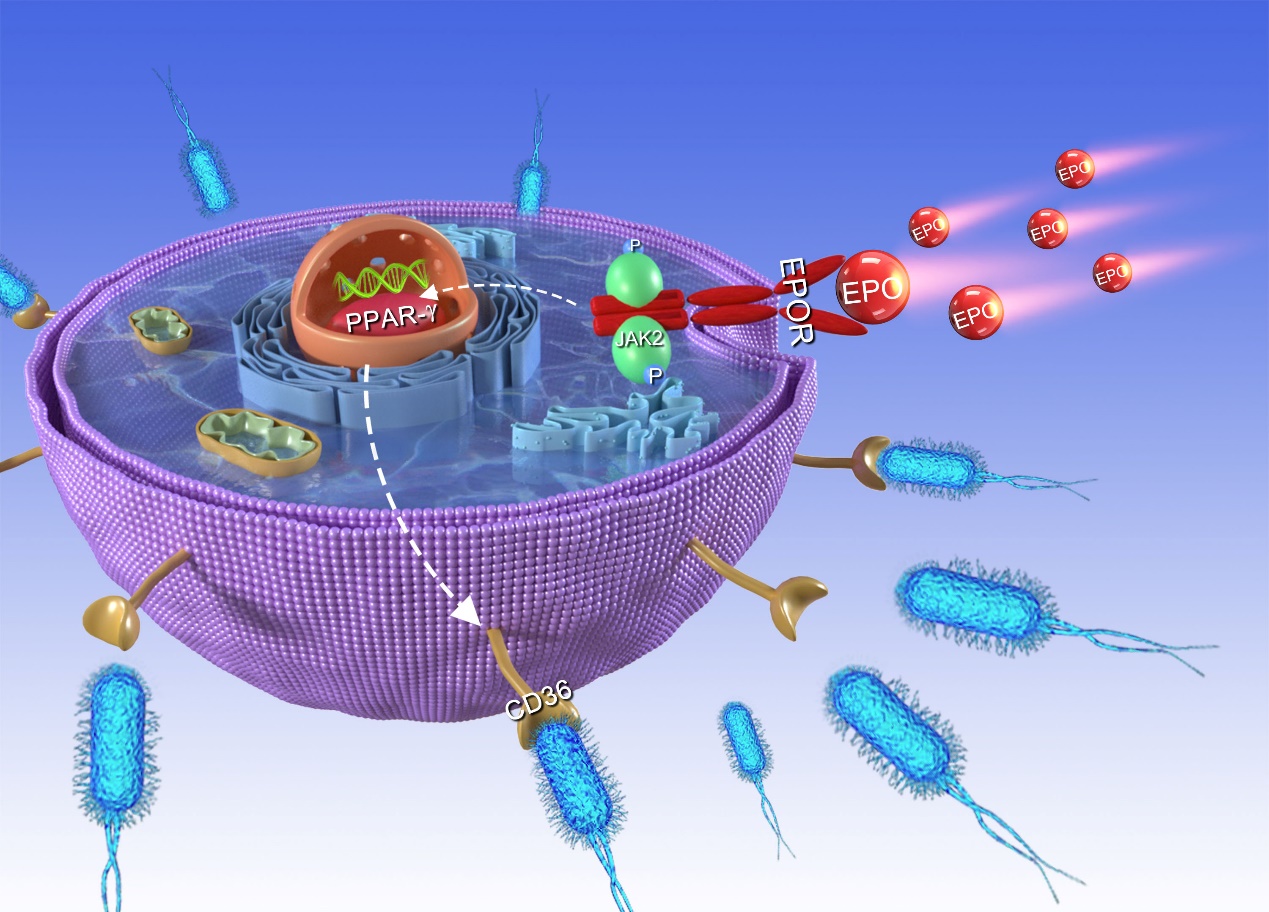


**Figure S8. Schematic diagram of EPO in macrophage clearance of bacteria.**

Following *E. coli* infection, macrophage EPO and EPOR levels were increasingly induced. Activation of EPO signaling enhanced macrophage phagocytosis of *E. coli* via peroxisome proliferator-activated receptor γ (PPARγ)-induced CD36.
